# Supplementary material for: Antibiotic thermorubin tethers ribosomal subunits and impedes A-site interactions to perturb protein synthesis in bacteria
Source: Nat Commun. 2023 Feb 17;14:918. doi: 10.1038/s41467-023-36528-7 (PMC9938272; doi:10.1038/s41467-023-36528-7)
Supplement: Supplementary file 1 — Supplementary Information [file 41467_2023_36528_MOESM1_ESM.pdf]

Supplementary Information

Antibiotic Thermorubin Tethers Ribosomal Subunits and Impedes A-site Interactions to Perturb Protein Synthesis in Bacteria

Narayan Prasad Parajuli, Andrew Emmerich, Chandra Sekhar Mandava, Michael Y. Pavlov, Suparna Sanyal*

*Corresponding author: suparna.sanyal@icm.uu.se

This file contains:

Supplementary Figures (1-21)

Supplementary Table (1)

Supplementary Notes (1-3)

Supplementary Figures

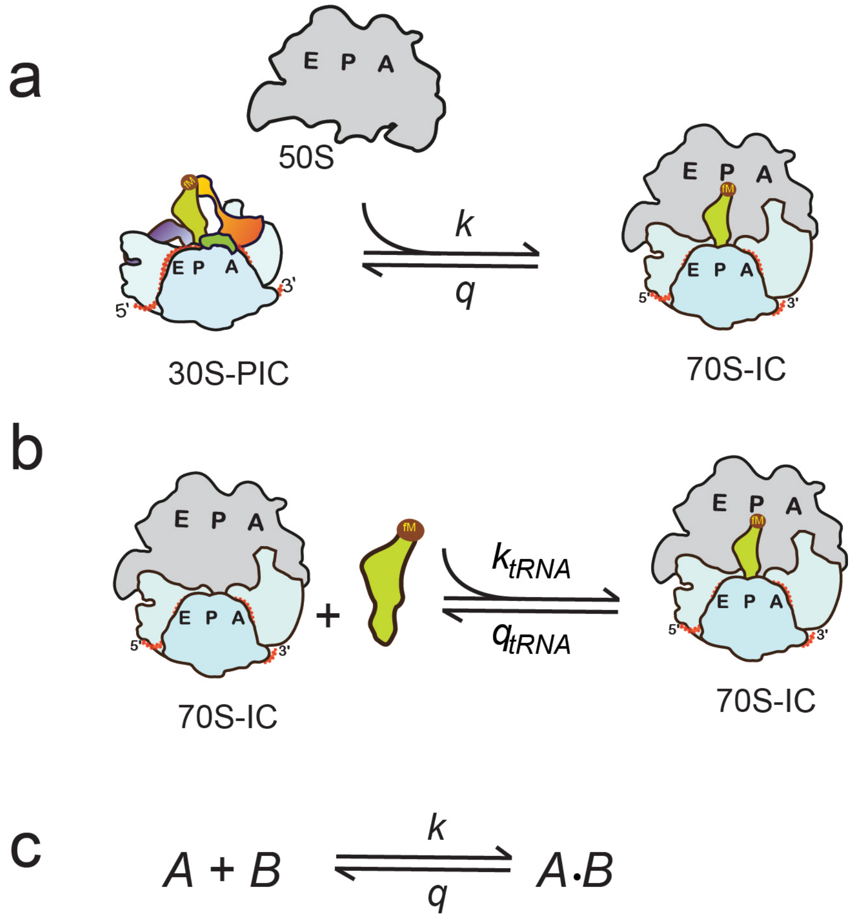

Supplementary Fig. 1. Initiation complex formation. (a) Formation of 70S initiation complex (70S-IC) by association of the 50S subunit with complete 30S pre-IC (30S-PIC) containing three initiation factors IF1, IF2, IF3 plus mRNA and fMet-tRNA^{fMet}, all bound to the 30S subunit. (b) Formation of 70S initiation complex (70S-IC) by direct binding of BOP•Met-tRNA to mRNA-programmed 70S ribosome. (c) Formation of generic $A*B$ complex from A and B moieties.

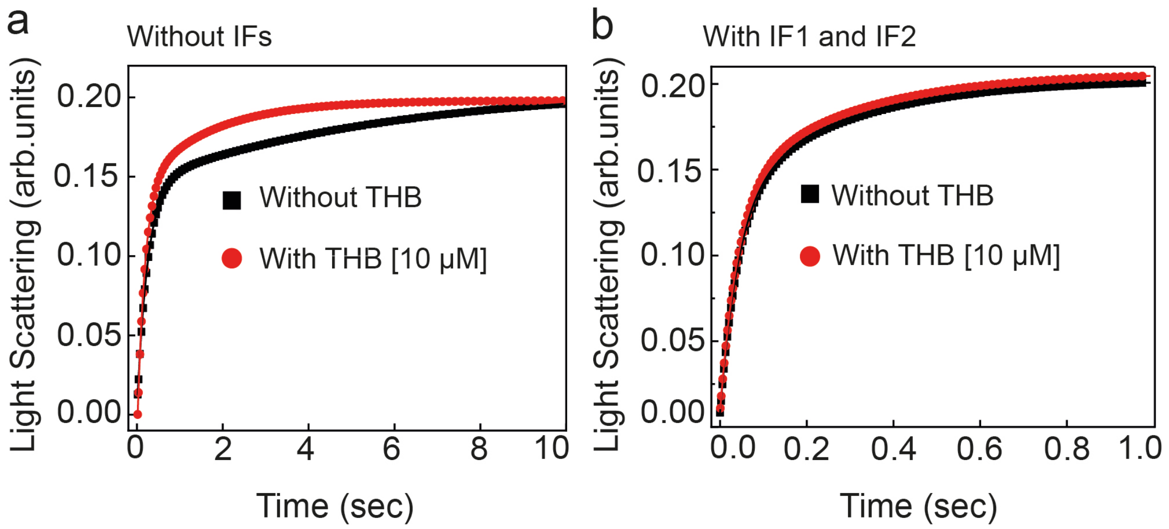

Supplementary Fig. 2. Effects of THB on ribosomal subunit association with and without IFs.

(a) Time course of 70S ribosome formation upon mixing 50S subunit with naked 30S subunit in the absence (black trace) and presence (red traces) of thermorubin (THB) (added to both 50S and 30S subunits). (b) Time courses of 70S IC formation upon mixing 50S subunit with 30S pre-IC without IF3 (30S:IF1:IF2:fMet-tRNA^{fMet}:mRNA) in the absence (black trace) and presence (red traces) of THB. The mixing was done in a stopped-flow instrument and Rayleigh light scattering at 365 nm was monitored. Experiments were conducted in triplicates and average data are plotted. Solid lines in (a) and (b) are the fits of data to complex formation equation (Supplementary Eq. (9)).

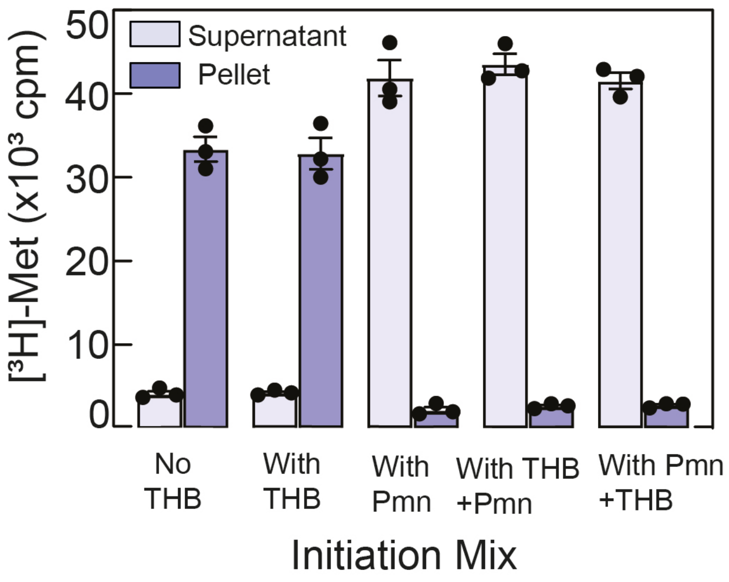

Supplementary Fig. 3. Effects of THB on the extent of fMet-tRNA^{fMet} binding to mRNA-programmed 70S IC and puromycin reactivity of the bound fMet-tRNA^{fMet}.

70S IC was formed by incubating 70S ribosomes with XR7 mRNA coding for Met-Phe-Phe-Stop, f[^{3}H]Met-tRNA^{fMet}, IF1, IF2, and IF3 to which Puromycin (Pmn) [0 or 200 μM] was added for peptidyl transfer. The reaction was quenched with 50% HCOOH and the [^{3}H]Met counts were measured from both the supernatant and pellet fractions. THB was either pre-incubated with 70S ribosomes or added simultaneously with mRNA, IFs, and f[^{3}H]Met-tRNA^{fMet} as indicated. The bars represent amounts of f[^{3}H]Met-tRNA^{fMet} retained in the ribosomal pellet or released into supernatant ([^{3}H]Met-Pmn) in the absence or presence of THB. Error bars are SEM of data.

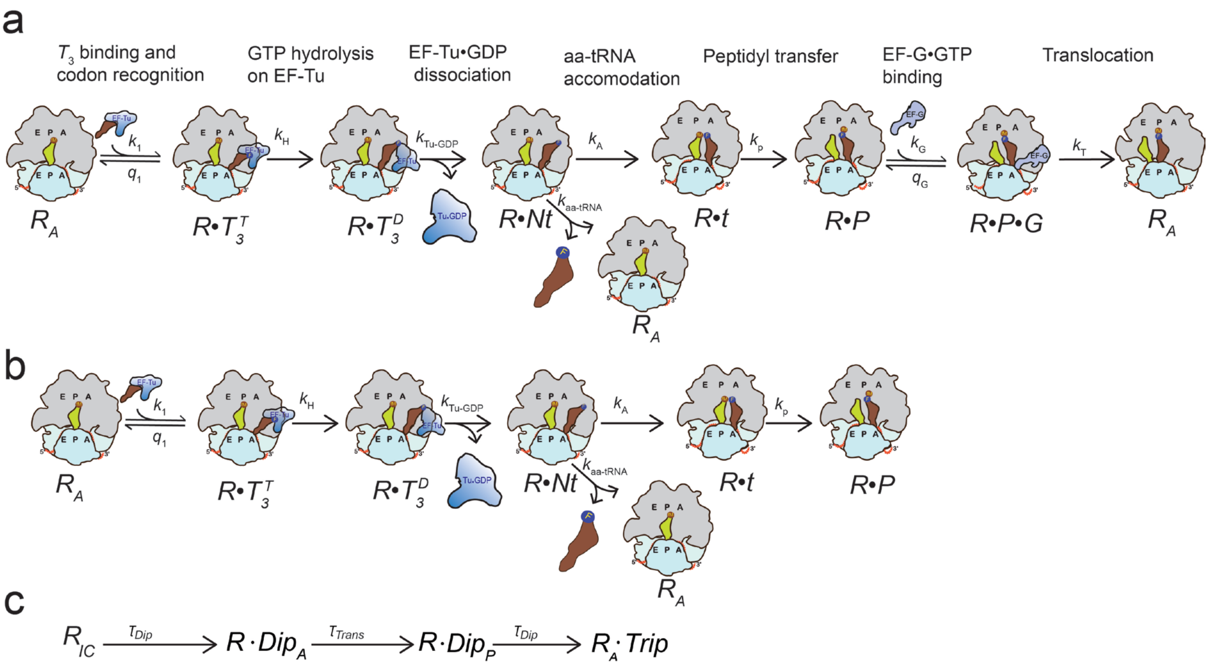

Supplementary Fig. 4. Elongation phase of translation. (a) Complete elongation cycle of the ribosome that starts with the binding of a ternary complex T_3^T containing aa-tRNA, EF-Tu, and GTP (superscript T means GTP form of EF-Tu) to a post-translocation ribosome (denoted here as R_A) containing an mRNA codon in otherwise vacant A-site. This binding results in the formation of $R \cdot T_3^T$ complex which is converted to $R \cdot T_3^D$ complex (superscript D means Tu in GDP form) upon codon decoding by aa-tRNA anticodon and subsequent GTP hydrolysis on EF-Tu with rate k_H. From $R \cdot T_3^D$ complex Tu·GDP dissociates fast with the rate constant $k_{Tu \cdot GDP}$ leading to a complex denoted here as $R{\cdot}Nt$ with non-accommodated aa-tRNA in the A-site of 30S subunit. This aa-tRNA either accommodates with rate k_A into the A-site on the 50S subunit leading to a complex denoted here as $R{\cdot}t$ or dissociates with the rate $k_{aa\text{-}tRNA}$ from the ribosome. The accommodated A-site aa-tRNA accepts a peptide from the P-site tRNA in a peptidyl transfer (PT) reaction that occurs with rate k_p leading to complex $R{\cdot}P$ in which a newly formed A-site peptidyl-tRNA carries a peptide extended by one amino acid. To this $R{\cdot}P$ complex EF-G (G) binds forming complex $R{\cdot}P{\cdot}G$. The bound EF-G makes the new peptidyl-tRNA translocate from the A- to P-site of the ribosome with rate k_T leading back to the ribosome R_A with extended peptide and a new A-site codon in otherwise empty A-site ready to bind a new ternary complex. (b) Curtailed, "single cycle" version of the complete elongation cycle depicted in (a) that ends at the $R{\cdot}P$ complex due to EF-G omission (describes a "dipeptide formation experiment"). (c) Simplified, two-amino acid addition reaction that requires two elongation cycles depicted in (a).

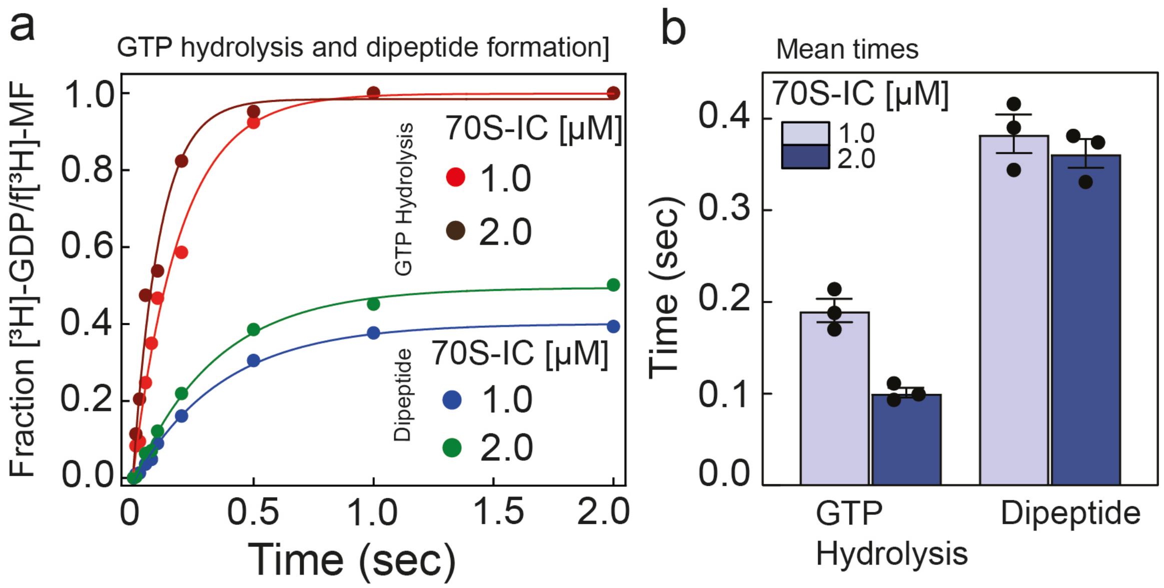

Supplementary Fig. 5. Dependence of the rates of GTP hydrolysis and dipeptide formation in THB presence on 70S IC concentration. (a) Time course of GTP hydrolysis and dipeptide formation after mixing 0.5 μM ternary complexes (T_3) EF-Tu•[^{3}H]GTP•Phe-tRNA^{Phe} with 1 or 2 μM 70S-IC pre-incubated with 10 μM THB. (b) Mean times of GTP hydrolysis on EF-Tu (τ_{GTP}) and dipeptide formation (τ_{Dip}) were estimated from the fit of data in (a) with single exponential (Supplementary Eq. (38)) and double exponential (Supplementary Eq. (37)) functions, respectively. Error bars in (b) are SEM of data.

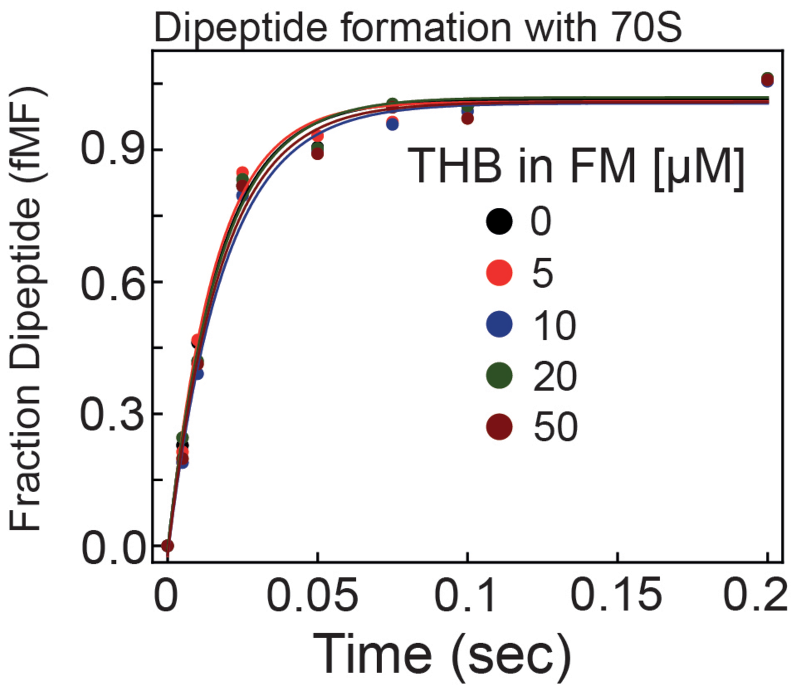

Supplementary Fig. 6: Kinetics of dipeptide formation with THB added in factor mix. Time course of dipeptide formation upon mixing of 0.5 μM 70S IC with the factor mix (FM) containing 5 μM EF-Tu•GTP•Phe-tRNA^{Phe} ternary complexes and THB in indicated concentrations. Solid lines are double exponential fits of the data (Supplementary Eq. (37)). Experiments were conducted in triplicates and average data are plotted.

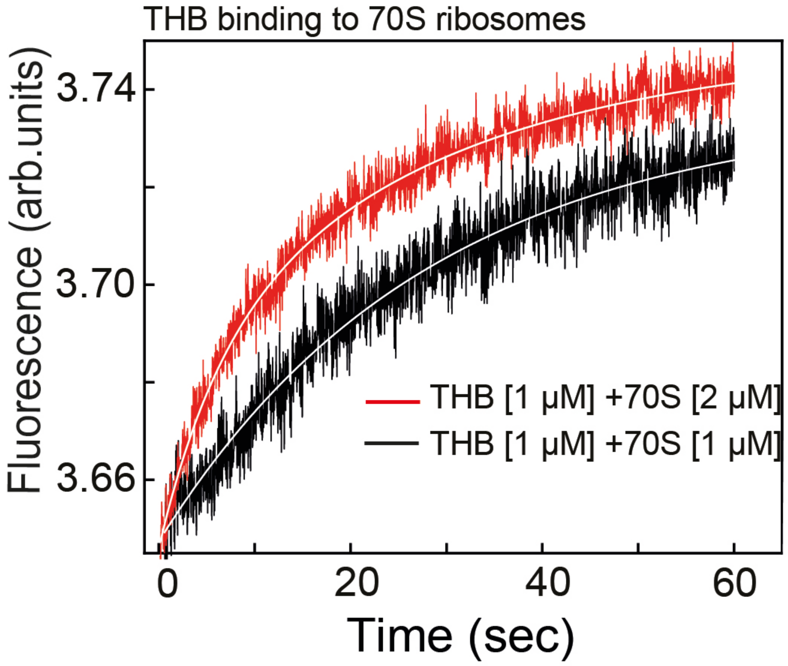

Supplementary Fig. 7. Binding of THB to the vacant 70S ribosomes. Time course of THB (1 μM) binding to 70S ribosome (black 1 μM and red 2 μM), monitored in stopped-flow instrument by the increase in THB fluorescence at 340 nm. The solid lines represent fit of the data to Supplementary Eq. (8) (red trace) and Supplementary Eq. (9) (black trace) that describe "complex formation".

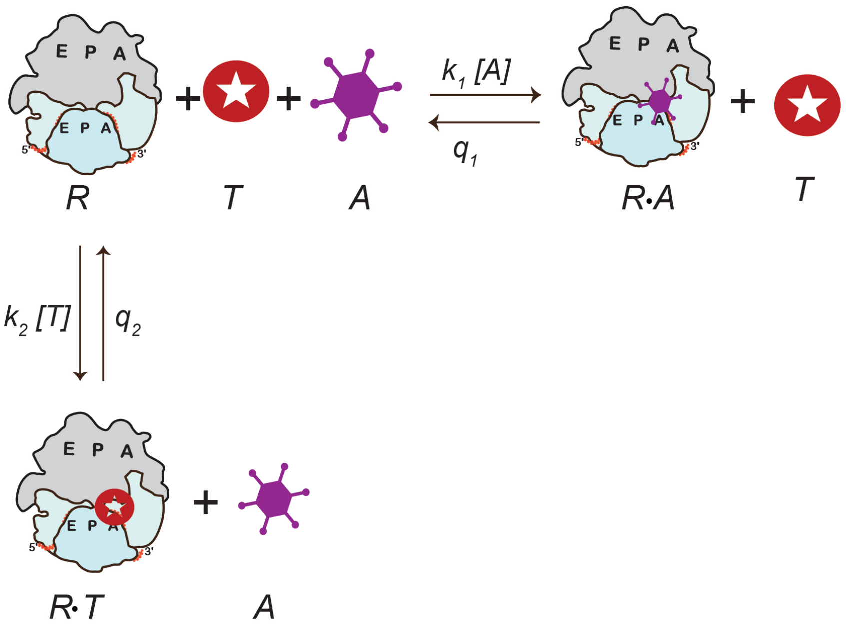

Supplementary Fig. 8. Kinetic scheme of chase experiment. Ribosome R can either bind Arbekacin (A) with association rate constant k_1 and dissociation rate constant q_1 or Thermorubin (T) with association rate constant k_2 and dissociation rate constant q_2 and their binding is mutually exclusive. The chase reaction starts with the addition of Arbekacin to a pre-formed $R*T$ complex after which the disappearance of the $R*T$ complex (that slowly converts to the $R*A$ complex) is monitored by changing Thermorubin fluorescence.

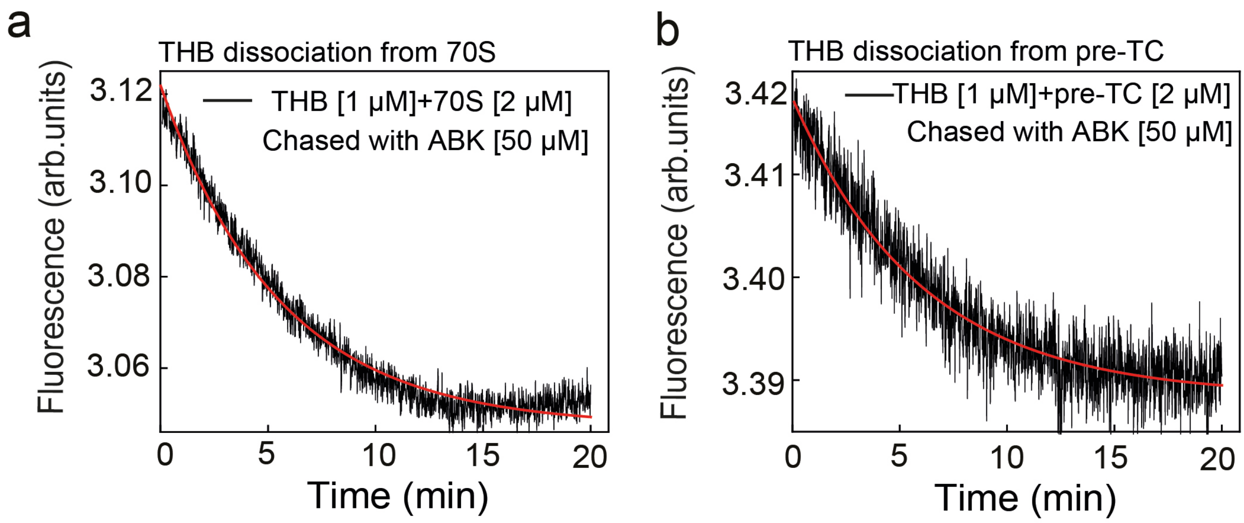

Supplementary Fig. 9. Dissociation of THB from vacant 70S ribosomes or pre-termination (pre-TC) complex measured in chase experiment. In order to follow the dissociation of THB from the ribosome we reacted THB-bound 70S ribosome (a) or pre-TC (b) with a huge excess of aminoglycoside arbekacin. The decreasing signal of fluorescence (340 nm) indicates the dissociation of THB from the A site chased by arbekacin. Solid lines are single exponential fit of the data (Supplementary Eq. (19)).

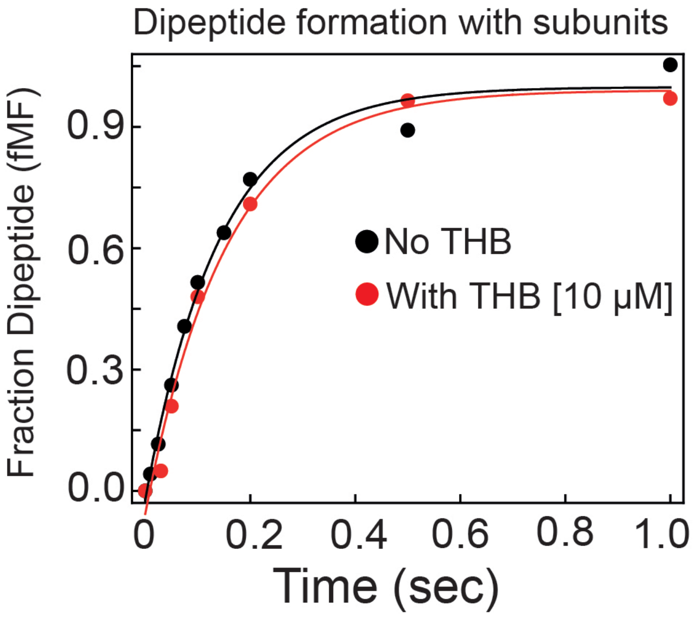

Supplementary Fig. 10. Effect of THB on the kinetics of dipeptide formation starting from 30S pre-IC. Time course of dipeptide formation when the reaction was started by adding 30S pre-IC containing 30S, XR7 mRNA, f[^{3}H]Met-tRNA^{fMet}, and all three IFs to a mix containing 50S subunits and ternary complex (T_3) (5 µM), with or without THB (10 µM) added to both mixes. Solid lines are 'double exponential' fits of the data to the equation of a two-step reaction (Supplementary Eq. (37)). Experiments were conducted in triplicates.

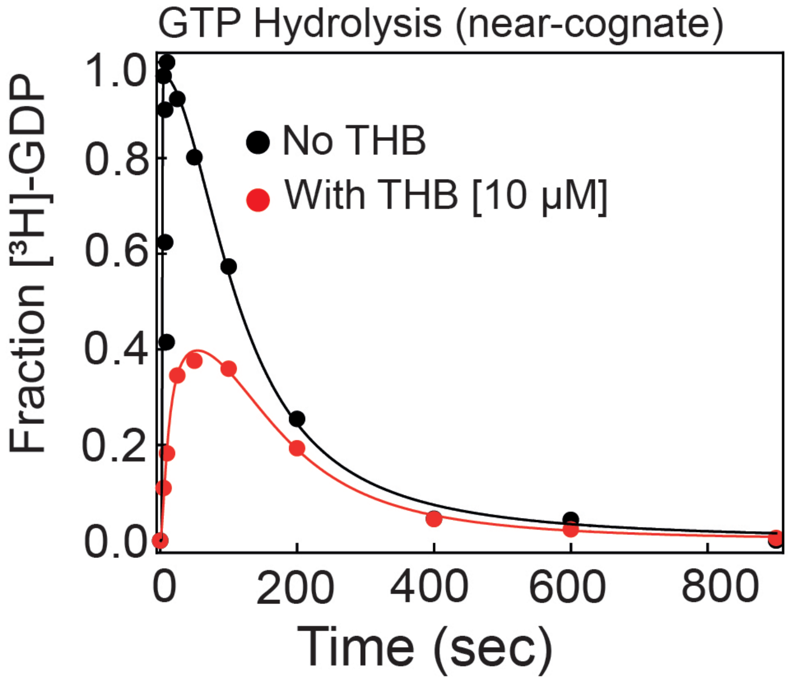

Supplementary Fig. 11. Effect of THB on the kinetics of GTP hydrolysis on EF-Tu with a near-cognate codon in the ribosomal A-site. Time course of GTP hydrolysis on EF-Tu•[^{3}H]GTP•Phe-tRNA^{Phe} ternary complex (0.3 μM) after mixing with 1 μM 70S IC programmed with near-cognate mRNA codon (CUC) in the A-site. The reaction proceeded in the absence (black) and presence (red) of THB (added to both mixes during pre-incubation). Solid lines are double exponential fits of the data. The rate constant of the first exponent here corresponds to the rate of GTP hydrolysis while the rate constant (0.36 min^{-1}) of the second exponent corresponds to slow spontaneous GDP exchange on EF-Tu•GDP in the absence of EF-Ts.

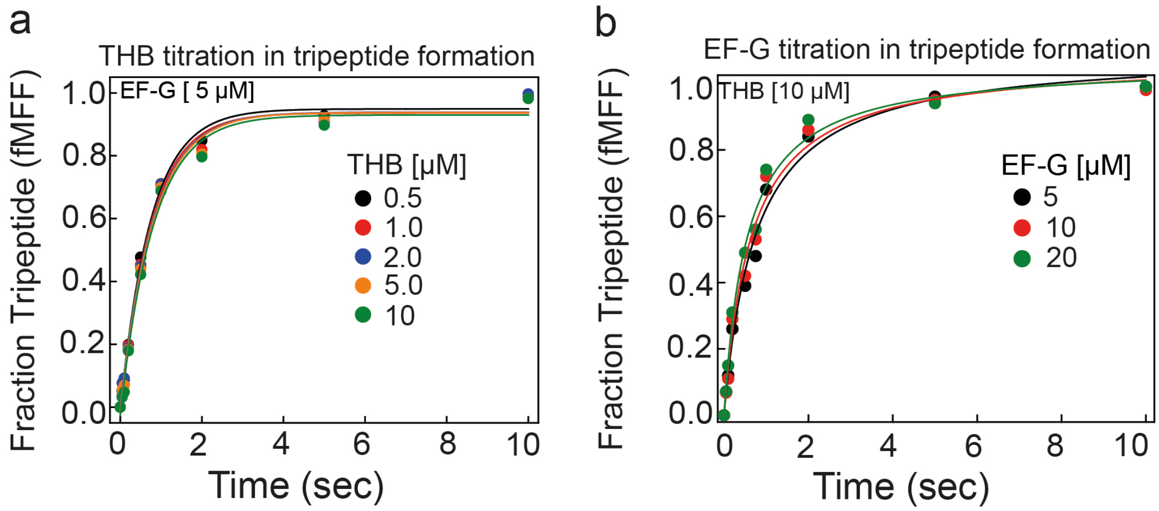

Supplementary Fig. 12. Effect of variation of THB or EF-G concentrations on the kinetics of tripeptide formation. (a) Time courses of tripeptide (fMet-Phe-Phe) formation with 10 μM ternary complex (T_3) and 5 μM EF-G. 70S IC programmed with fMFF-stop mRNA was pre-incubated with indicated THB concentrations before mixing in the quench-flow instrument. (b) The same as (a) but 10 μM THB concentration was kept constant and EF-G concentration was varied as indicated. Experiments were conducted in duplicates and the data was fitted to a "three-step kinetic scheme" (Supplementary Eq. (51)).

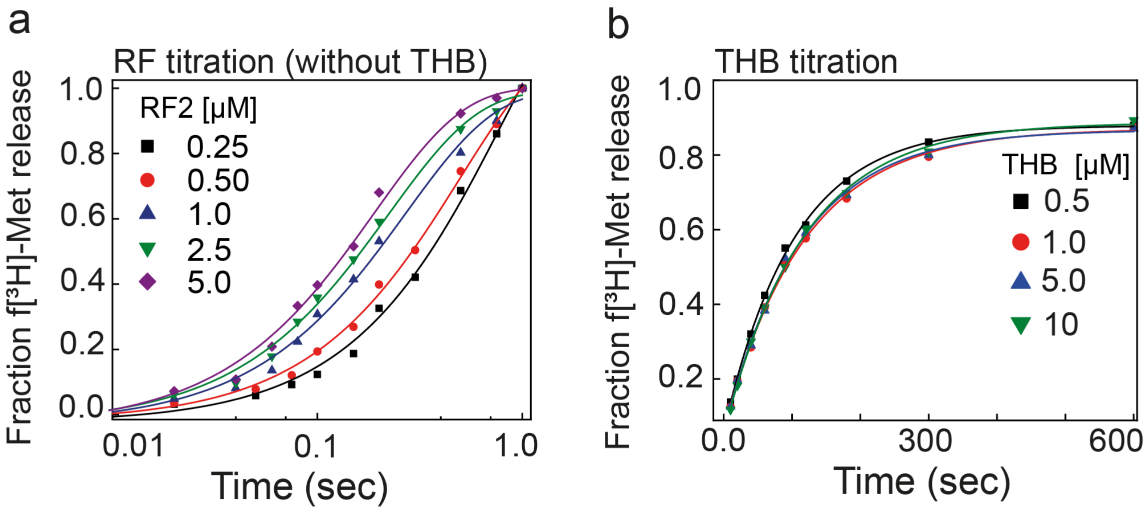

Supplementary Fig. 13. Rate of f[^{3}H]Met release by RF2 at different RF2 and THB concentrations. (a) Kinetics of f[^{3}H]Met release from the P-site f[^{3}H]Met-tRNA^{fMet} of pre-TC (0.5 μM) in the absence of THB, by RF2 added in indicated concentrations. (b) Kinetics of f[^{3}H]Met release from the P site of pre-TC (0.5 μM) by RF2 (5 μM) with increasing concentrations of THB (0.5 to 10 μM). Experiments in (a) and (b) were conducted in triplicates and the averaged data are plotted. Solid lines in (a) and (b) are single exponential fits of the data.

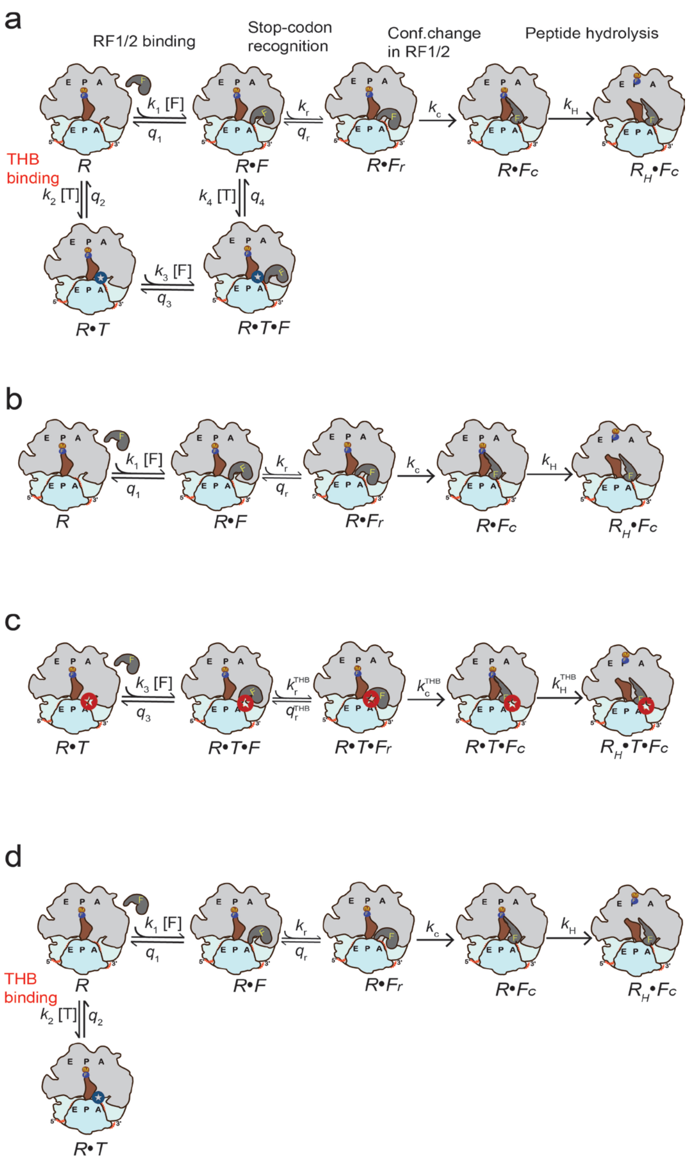

Supplementary Fig. 14. Termination phase of translation. (a) Termination in the presence of Thermorubin (THB). Here, terminating ribosome with a stop codon in the A-site (denoted as R here) binds release factor (denoted as F) with rate constant k_1 forming release factor pre-bound complex $R{\cdot}F$ from which F can either dissociate with rate constant q_1 or recognize stop codon with rate constant k_r forming complex $R{\cdot}F_r$. Complex $R{\cdot}F_r$ can either return back to $R{\cdot}F$ with date constant q_r or proceed forward with rate k_c to complex $R{\cdot}F_c$ in which release factor F has undergone a conformation change that leads to the ester bond hydrolysis in peptidyl-tRNA with rate constant k_H and peptide release. THB (T) can bind both to R forming complex $R{\cdot}T$ and to $R{\cdot}F$ forming complex $R{\cdot}T{\cdot}F$. These THB binding reactions are described by rate constants k_2, q_2, k_4, and q_4 as indicated. (b) Termination in Thermorubin (THB) absence (the same as (a) except that THB-containing complexes are omitted). (c) Termination for the case of persistent THB presence. The same as (b) except that THB is assumed to be bound to all complexes on the termination pathway. (d) Termination in the presence of THB. The same as (a) except that simultaneous THB and RF1/2 pre-binding to termination complex R is assumed impossible (i.e., it is assumed that $R{\cdot}T{\cdot}F$ complex cannot be reached).

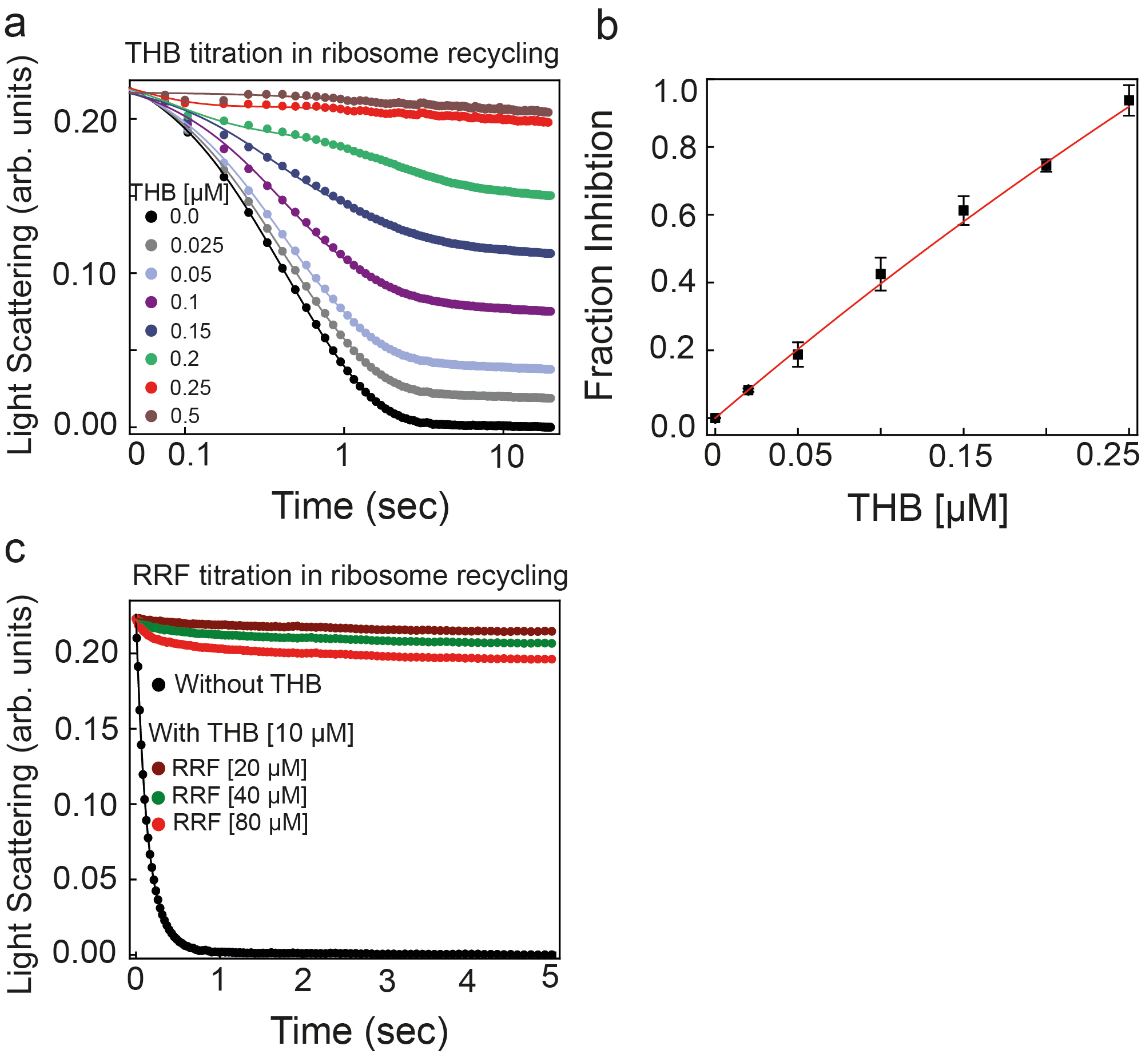

Supplementary Fig. 15. Effect of THB on the splitting of post-termination ribosome complexes (post-TC). (a) Post-TC (0.25 µM) was pre-incubated with indicated THB concentrations after which the time course of their splitting into subunits by RRF (20 µM), EF-G (10 µM), and IF3 (1 µM) was monitored by Rayleigh light scattering at 365 nm in stopped-flow. (b) Dependence of the fraction of non-split ribosomes on THB concentration in (a); solid line here is the linear fit of the data with error bars indicating SEM of the data. (c) Post-TC (0.25 µM) was pre-incubated with 10 µM THB after which the time course of their splitting into subunits by RRF (added in indicated concentrations), EF-G (10 µM), and IF3 (1 µM) was monitored in stopped-flow. Solid lines in (a) and (c) represent the double exponential fits of the data. Experiments were conducted in triplicates.

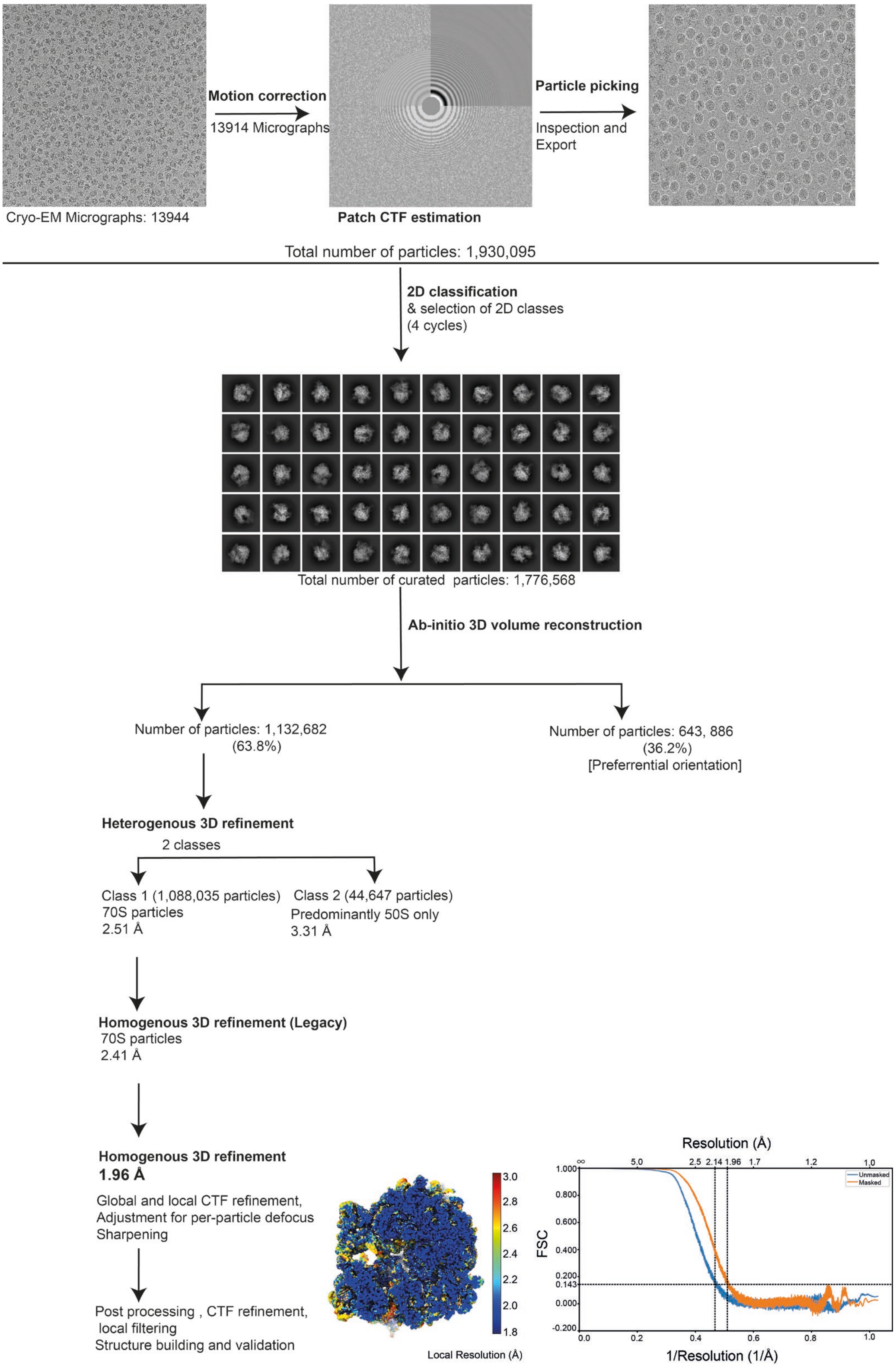

Supplementary Fig. 16. Pipeline for Cryo-EM data processing. Cryo-EM data was processed using CryoSPARC and the model building was performed using Coot. Fourier Shell Correlation (FSC) analysis is presented in the bottom right (blue – unmasked and orange–masked half maps). The final average resolution of the highly homogeneous THB-bound 70S IC, determined on the basis of the FSC value of 0.143 (orange curve) is 1.96 Å.

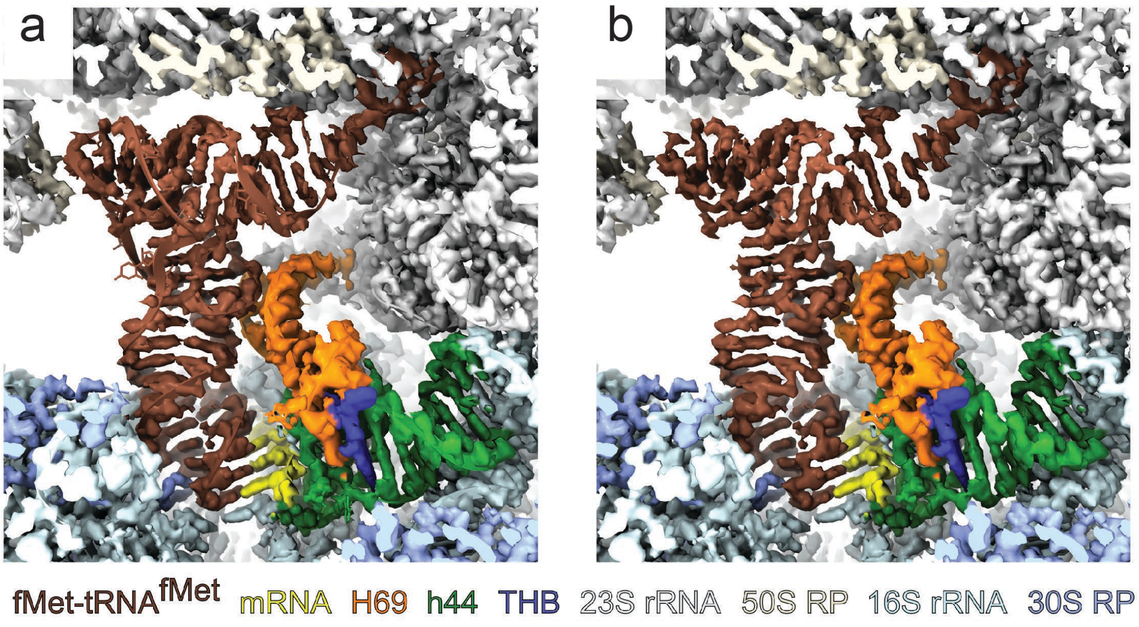

Supplementary Fig. 17. Close look into the THB binding site at the subunit interface. (a) Cryo-EM map fitted to model and (b) map without model depicting bound THB (blue) at the interface between H69 (orange) and h44 (green) of 23S and 16S rRNA respectively. Bound fMet-tRNA^{fMet} (saddle brown) and mRNA (yellow) are also seen clearly. The same contour level was used throughout the figures including tRNA, mRNA, rRNA, and THB, which shows the matching density of all elements in the THB binding pocket of the ribosome.

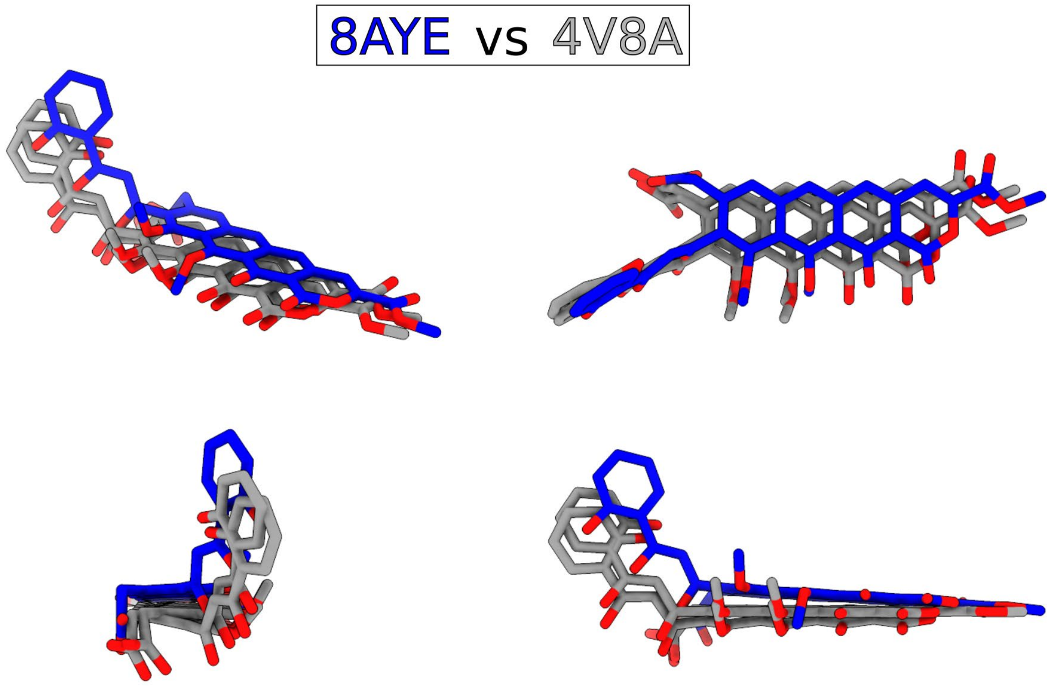

Supplementary Fig. 18. Comparison of our cryo-EM structure of *E. coli* ribosome-bound THB in the presence of P-site tRNA (8AYE, blue) with the earlier crystal structure of THB-70S (*T. thermophilus*) complex (4V8A, grey). The superposition of the two structures in four different orientations shows slightly different conformation of the orthohydroxyphenyl group of THB. The difference can be due to THB binding to functional (current) vs. non-functional (earlier) ribosomal complex. The difference can also be due to difference of the ribosomes from the two bacterial species.

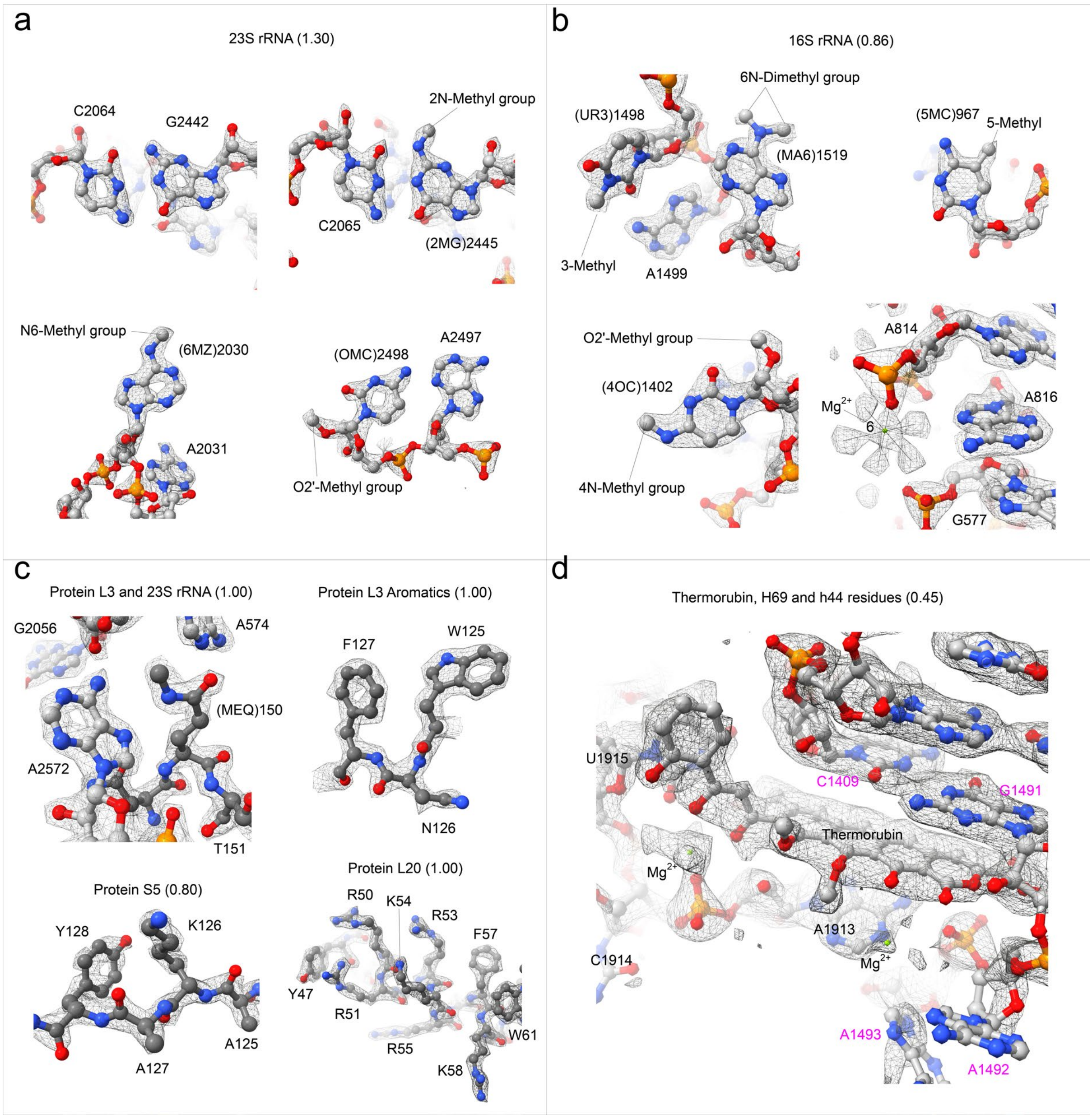

Supplementary Fig. 19. Illustration depicting map-quality of cryo-EM reconstruction of THB bound 70S ribosome (this study) (PDB 8AYE). (a-b) Examples of modifications of rRNA bases in both 23S and 16S rRNA are shown as sticks with overlaid EM densities shown as mesh. In (b) close-up density for Mg^{++} with coordination of water/oxygen molecules can be seen. (c) Example EM-densities showing side chain conformations of amino acids in ribosomal proteins including L3, L20, and S5. (d) EM-density of THB binding site with surrounding residues and Mg^{++}. The numbers in the brackets indicate the map threshold.

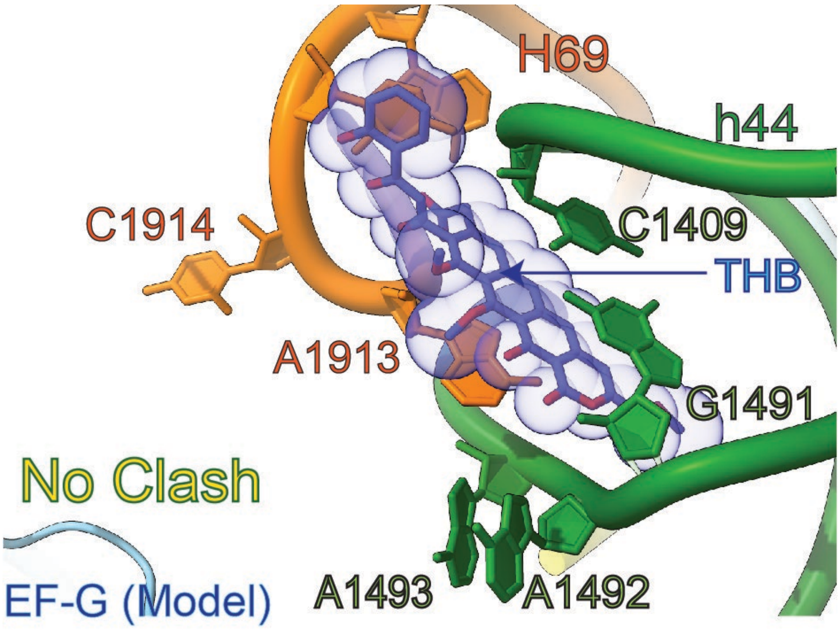

Supplementary Fig. 20. Superimposition of THB-bound 70S ribosome (this study) with the EF-G bound pre-translocation ribosome (PDB 7SSL). Overlay of these two structures shows no steric hindrance for EF-G binding in the presence of THB in the ribosomal A site.

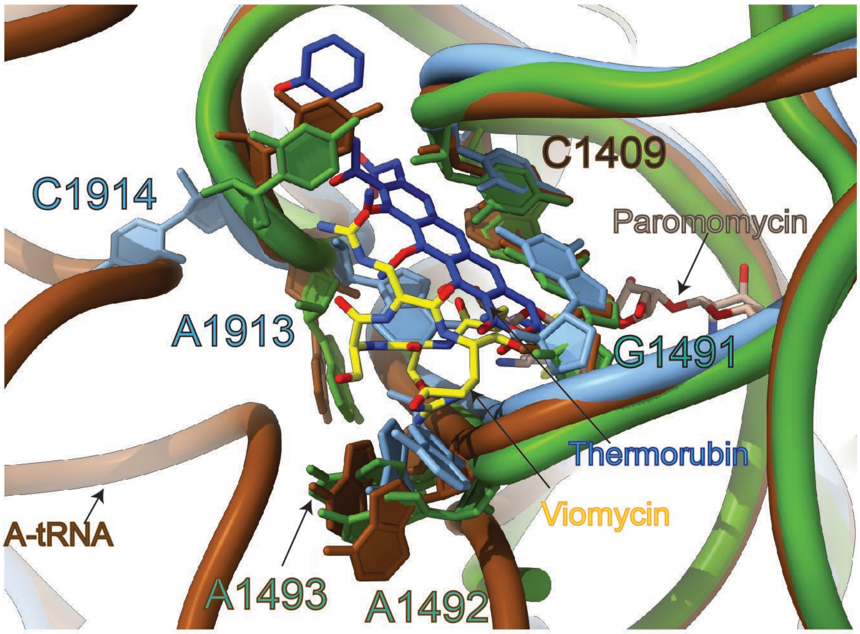

Supplementary Fig. 21. Close-up view of the A-site of 70S ribosome bound to thermorubin (blue) (PDB 8AYE), aminoglycoside paromomycin (brown) (PDB 7K00), and tuberactinomycin viomycin (yellow) (PDB 6LKQ). Important 16S and 23S rRNA nucleobases interacting with these antibiotics are displayed**.**

Supplementary Table 1: **Data collection, refinement, and validation statistics for THB-bound mRNA-programmed 70S-fMet-tRNA^{fMet} complex**

**Data Collection**	
Microscope	Titan Krios
Camera	Gatan K2
Voltage (kV)	300
Magnification	105,000
Pixel Size at Detector (Å)	0.8617
Defocus range (μM)	-0.6 to -2.0
Total dose (e/Å^2)	30.745
Dose per frame (e/Å^2)	1.025
Number of frames	30
**Image Processing**	
Number of images	13944
Particles used for 3D reconstruction	1,088,035
Resolution (Global, Å) FSC 0.143 Masked	1.96
Resolution (Global, Å) FSC 0.143 Unmasked	2.14
Resolution (Global, Å) FSC 0.5 Masked	2.00
Resolution (Global, Å) FSC 0.5 Unmasked	2.20
**Model Building and Refinement**	
Bond RMS deviation - Length (Å) (# > 4σ)	0.008 (1)
Bond RMS deviation - Angles (°) (# > 4σ)	0.905 (42)
CC (mask)	0.89
CC (box)	0.75
**Validation**	
MolProbity score	1.42
Clash score	4.43
Ramachandran Outliers (%)	0.11
Ramachandran Allowed (%)	2.63
Ramachandran Favored (%)	97.26
Rotamer outliers (%)	1.20
Cβ outliers (%)	0.00

Supplementary Note 1: Initiation and Complex formation kinetics

The initiation stage of translation cycles starts with the formation of 30S pre-initiation complex (30S pre-IC), which involves binding of IF1, IF2, and IF3 plus mRNA followed by fMet-tRNA^{fMet} binding. Thus, 30S pre-IC contains all three initiation factors and a P-site bound fMet-tRNA^{fMet} that interacts with AUG codon of mRNA in the P-site of the 30S subunit [1,2]. Formation of a 70S ribosome or 70S initiation complex (70S IC) occurs by the association of 50S subunit with naked 30S subunit or complete 30S pre-IC (30S-PIC), respectively [1,3] (as illustrated in Supplementary Fig. 1a). The complete 30S pre-IC is used in the experiments in Figure 2a, while 30S pre-IC formed without IF3 is used in the experiments shown in Supplementary Fig. 2.

fMet-tRNA^{fMet} can also bind directly to mRNA programed 70S ribosomes (as in experiments in Figs. 2c and d) through a "non-orthodox" initiation pathway [4]. Such a binding is described by the kinetic scheme shown in Supplementary Fig. 1b. The kinetic schemes shown in Supplementary Figs. 1a and 1b can be viewed as particular cases of a generic "complex formation" scheme in Supplementary Fig. 1c. The kinetic equations that govern the dynamics of this generic "complex formation" scheme (Supplementary Fig. 1c) are discussed below.

Kinetics of complex formation and dissociation.

Denoting A·B as C the differential equation describing the dynamics of C-concentration in Supplementary Fig.1c is:

$$\frac{d}{dt}c = k\cdot a\cdot b - q\cdot c = k\{(a_0-c)(b_0-c)-K\cdot c\} \tag{1}$$

Here a_0 and b_0 are total (free plus in C-complex) while a and b are current concentrations of A and B; c is the current concentration of complex C and $K=q/k$ is the equilibrium dissociation constant of complex formation. The above equation can be re-written as:

$$k(p_0-r_0)dt = \frac{dc}{(r_0-c)} - \frac{dc}{(p_0-c)} \tag{2}$$

Here, r_0 and p_0 are the two roots of quadratic polynomial of c:

$$y(c) = (a_0-c)(b_0-c)-K\cdot c = c^2-(a_0+b_0+K)\cdot c + a_0 b_0 \tag{3}$$

Integrating Supplementary Eq. (2) one obtains (assuming that $r_0 > p_0$) :

$$c(t) = \frac{p_0 - r_0\Omega\exp(-k(r_0-p_0)t)}{1-\Omega\exp(-k(r_0-p_0)t)} \tag{4}$$

Here we denoted:

$$\Omega = (p_0 - c(0))/(r_0-c(0)) \tag{5}$$

In the case when one studies complex formation by mixing A and B at time zero, c(0)=0 so that $\Omega = p_0/r_0$. Using this, Supplementary Eq. (4) simplifies to:

$$c(t) = p_0\frac{1-\exp(-k(r_0-p_0)t)}{1-(p_0/r_0)\exp(-k(r_0-p_0)t)} \tag{6}$$

In the case when $a_0 >> b_0$ and $K >> b_0$ the roots r_0 and p_0 can be readily estimated and Supplementary Eq. (6) reduces further to a well -known equation for complex formation kinetics:

$$c(t) \approx b_0\frac{a_0}{a_0+K}\{1-\exp(-(k\cdot a_0+q)t)\} \tag{7}$$

It shows that by fitting $c(t)$ to an exponential function at different a_0 values (initial A-concentrations) one can recover both the association and dissociation rate constants *k* and *q*, respectively[5].

When *K* is very small in comparison with both a_0 and b_0, as in all association experiments in Fig. 2 and Supplementary Fig. 2, the r_0 and p_0 roots approach a_0 and b_0, respectively. In this case:

$$c(t) \approx b_0 \frac{1-\exp(-k(a_0-b_0)t)}{1-(b_0/a_0)\exp(-k(a_0-b_0)t)} \tag{8}$$

If, in addition $a_0 \approx b_0$, the above relation further simplifies to [3]:

$$c(t) \approx b_0 \frac{kb_0 \cdot t}{1+kb_0 \cdot t} \tag{9}$$

Kinetic of complex dissociation in THB "chase "experiment

The kinetics of THB chase experiment can be described by a kinetic scheme shown in Supplementary Fig. 8. Here, Thermorubin (THB), denoted as *T* in the figure, is first pre-incubated with a small excess of ribosomes (denoted as R here) to assure the formation of *R·T* complex. It can also be assumed (from measured equilibrium dissociation constant of *R·T* formation) that nearly all THB is bound in this *R·T* complex. The chase experiment in Supplementary Fig. 9 starts with the addition of a high concentration of another antibiotic, Arbekacin (denoted as A in Supplementary Fig. 8) that competes with THB for the same binding site on the ribosome [6]. One then monitors the dissociation of THB from ribosome as a nearly exponential reduction of fluorescence (Supplementary Fig. 9) because the re-binding of dissociated THB back to the ribosome is blocked by fast Arbekacin binding to the THB –free ribosomes. The formation and dissociation of *R·T* and *R·A* complexes is governed by two differential equations:

$$\begin{aligned} \frac{d}{dt}c_{RA} &= k_1[A]c_R - q_1 c_{RA} \\ \frac{d}{dt}c_{RT} &= k_2[T]c_R - q_2 c_{RT} \end{aligned} \tag{10}$$

Here, c_{RA} and c_{RT} are the current concentrations of *R·A* and *R·T* complexes, respectively; k_1 and q_1 are the association and dissociation rate constants for Arbekacin binding, respectively; k_2 and q_2 are those for Thermorubin binding. The time evolution of drug-unbound ribosome concentration c_R and free THB can be deduced from conservation laws for ribosomes and THB, respectively:

$$\begin{aligned} c_R &= c_{tot} - c_{RA} - c_{RT} \\ [T] &= [T]_{tot} - c_{RT} \end{aligned} \tag{11}$$

To obtain the kinetic of *R·T* concentration we use that k_1 and q_1 for Arbekacin are larger than k_2 and q_2 for THB and, in addition, that the Arbekacin concentration is much larger than that of THB. This implies that *R·A* complex equilibrates with free ribosomes very fast (on the time scale of THB dissociation) so that the following relation between the current *R* and *R·A* concentrations is valid:

$$c_R = K_1 \frac{c_{RA}}{[A]} \tag{12}$$

From this and the 'matter conservation law' applied to the ribosomes, it follows that:

$$c_R + c_{RA} = c_{RA} + K_1 \frac{c_{RA}}{[A]} = c_{RA}(1 + K_1 / [A]) = c_{tot} - c_{RT}$$

$$c_{RA} = (c_{tot} - c_{RT}) / (1 + K_1 / [A]) \tag{13}$$

$$c_R = K_1 \frac{c_{RA}}{[A]} = \frac{K_1}{[A]} \frac{(c_{tot} - c_{RT})}{(1 + K_1 / [A])}$$

Using the last expressions in Supplementary Eq. (13) and (11), one can re-write Supplementary Eq. (10) for complex $R{\cdot}T$ concentration as:

$$\frac{d}{dt} c_{RT} = vk_2 \left\{ ([T]_{tot} - c_{RT})(c_{tot} - c_{RT}) - w \cdot c_{RT} \right\} \tag{14}$$

Here, we introduced for compactness:

$$v = \frac{K_1}{[A] + K_1} \quad \text{and:} \quad w = \frac{q_2}{k_2 v} = K_2 \frac{K_1 + [A]}{K_1} \tag{15}$$

Following the same approach as in the previous section one obtains the following solution of Supplementary Eq. (14):

$$c_{RT}(t) = \frac{p_0 - r_0 \Omega \exp(-vk_2(r_0 - p_0)t)}{1 - \Omega \exp(-vk_2(r_0 - p_0)t)} \tag{16}$$

Here, r_0 and p_0 are the two roots of quadratic polynomial in c_{RT}:

$$y(c_{RT}) = ([T]_{tot} - c_{RT})(c_{tot} - c_{RT}) - w \cdot c_{RT} = c_{RT}^2 - ([T]_{tot} + c_{tot} + w)c_{RT} + [T]_{tot}\, c_{tot} \tag{17}$$

Like before (see Supplementary Eq. (6)) $\Omega = (p_0 - c_{RT}(0)) / (r_0 - c_{RT}(0))$ and $r_0 > p_0$. We will now use that at the start of experiment nearly all THB was ribosome bound, i.e., $c_{RT}(0) \approx [T]_{tot}$. After some simple algebra (and using that $p_0 r_0 = [T]_{tot}\, c_{tot}$) Supplementary Eq. (16) transforms to:

$$c_{RT}(t) = [T]_{tot} \frac{(c_{tot} - p_0) - (c_{tot} - r_0)\exp(-vk_2(r_0 - p_0)t)}{(r_0 - [T]_{tot}) - (p_0 - [T]_{tot})\exp(-vk_2(r_0 - p_0)t)} \tag{18}$$

In the chase experiment we use Arbekacin concentration sufficiently high to ensure that w in Supplementary Eq. (17) is much larger than the sum of total ribosome and THB concentrations. Under this condition and using that $w >> [T]_{tot}$, $w >> c_{tot}$, Supplementary Eq. (18) simplifies to:

$$c(t) \approx [T]_{tot} \frac{c_{tot} + (w + [T]_{tot})\exp(-Qt)}{(c_{tot} + w) + [T]_{tot}\exp(-Qt)} \approx \frac{w[T]_{tot}}{c_{tot} + w}\exp(-Qt) + \frac{c_{tot}[T]_{tot}}{c_{tot} + w} \tag{19}$$

Here:

$$Q = q_2 + \frac{K_1}{[A] + K_1} k_2 (c_{tot} + [T]_{tot}) \tag{20}$$

It shows that fitting experimental data on fluorescence decreases in Supplementary Fig. 9 to Supplementary Eq. (19) one obtains rate Q that is always larger than genuine THB

dissociation rate q_2. In other word, Q is the upper bound for q_2. Moreover, Supplementary Eq. (20) shows that the higher is the chaser A concentration the closer Q is to q_2.

Supplementary Note 2: Elongation phase of translation and its "mean-time" analysis

Elongation cycle of the ribosome is depicted in Supplementary Fig. 4a. The cycle starts with a post-translocation ribosome or with its mimic, a 70S Initiation complex (IC) containing an mRNA codon in otherwise vacant A-site (denoted here as R_A). To this ribosome R_A a ternary complex T_3^T contacting aa-tRNA, EF-Tu and GTP binds (superscript T means GTP form of EF-Tu) forming a $R \cdot T_3^T$ complex. Upon A-site codon recognition by aa-tRNA anti-codon in the decoding center of the ribosome GTP hydrolysis on EF-Tu in $R \cdot T_3^T$ complex occurs with the rate k_H leading to $R \cdot T_3^D$ complex (superscript D means Tu in GDP form). From this complex Tu·GDP dissociates fast with the rate constant $k_{Tu \cdot GDP}$ leading to a complex denoted here as $R{\cdot}Nt$ with non-accommodated aa-tRNA sitting in the A-site of the 30S subunit. Then aa-tRNA accommodates with rate k_A into the A-site on the 50S subunit leading to a complex denoted here as $R{\cdot}t$. During the accommodation aa-tRNA can dissociate with the rate $k_{aa\text{-}tRNA}$ from the ribosome in a so-called proofreading reaction also re-generating R_A (Supplementary Fig. 4a). When post-accommodation complex $R{\cdot}t$ is reached, the accommodated A-site aa-tRNA accepts the peptide from the P-site tRNA in a peptidyl transfer (PT) reaction that occurs with rate k_p leading to complex $R{\cdot}P$ in which a newly formed A-site peptidyl-tRNA carries a peptide extended by one amino acid. To this $R{\cdot}P$ complex EF-G (G) binds forming complex $R{\cdot}P{\cdot}G$. The bound EF-G makes the new peptidyl-tRNA to translocate from the A- to P-site of the ribosome with rate k_T leading back to the ribosome R_A with extended peptide and a new A-site codon in otherwise empty A-site ready to bind a new ternary complex. Importantly, EF-Tu· GDP dissociated from complex $R \cdot T_3^D$ requires GDP to GTP exchange factor EF-Ts to convert EF-Tu· GDP back to EF-Tu· GTP.

It should be noted that the elongation cycle in Supplementary Fig. 4a is somewhat simplified in that the step of T_3 pre-binding to the ribosome and the subsequent step of codon: anticodon recognition that leads to GTP hydrolysis on EF-Tu [7,8] are lumped here together. We have further assumed that after translocation EF-G dissociates from the post-translocated ribosome very fast so that a short-lived complex $R_A{\cdot}G$ can be omitted.

Single cycle kinetic experiments with elongating ribosome.

The ultimate aim of single cycle experiments is to determine the rate constants of kinetic schemes or, failing to determine all rate constants, to extract some of them or their combinations. The kinetic scheme of elongation cycle in Supplementary Fig. 4a can describe both multi-cycle and single cycle experiments depending, for example, on availability of T_3 with a cognate aa-tRNA able to read a new codon in the R_A ribosome (the

one to the right in Supplementary Fig. 4a) or availability of EF-G. A curtailed, “single cycle” version of elongation cycle for the case when EF-G is excluded is depicted in Supplementary Fig. 4b. In a corresponding single cycle experiment [9] one usually monitors the time course of accumulation of complex $R{\cdot}P$ that contains the extended peptide (dipeptide in case when R_A is a 70S initiation complex). After mixing R_A ribosomes and ternary complexes T_3^T at the start of experiment the ribosome R_A transits through several complexes as time passes by ending up in complex $R{\cdot}P$ (see Supplementary Fig. 4b) from which it cannot move further (because of EF-G omission). $R{\cdot}P$ accumulation can be monitored (with the help of HPLC) as the time course of the formation of peptide with the length increased by one amino acid [9].

The dynamic of concentrations of R_A ribosomes, different ribosomal complexes, free T_3^T, dissociated Tu·GDP and aa-tRNA in Supplementary Fig. 4b is governed by the following set of differential equations:

$$
\begin{aligned}
&\frac{d}{dt}c_{RA} = -k_1 c_{RA} c_{T3} + q_1 c_{RT} + k_{AA-tRNA} c_{RD} \\
&\frac{d}{dt}c_{T3} = -k_1 c_{RA} c_{T3} + q_1 c_{RT} \\
&\frac{d}{dt}c_{RT} = k_1 c_{RA} c_{T3} - (q_1 + k_H) c_{RT} \\
&\frac{d}{dt}c_{RD} = k_H c_{RT} - k_{Tu\cdot GDP} c_{RD} \\
&\frac{d}{dt}\left[Tu \cdot GDP\right] = k_{Tu\cdot GDP} c_{RD} \\
&\frac{d}{dt}c_{RNt} = k_{Tu\cdot GDP} c_{RD} - (k_A + k_{aa-tRNA}) c_{RNt} \\
&\frac{d}{dt}\left[aa - tRNA\right] = k_{aa-tRNA} c_{RNt} \\
&\frac{d}{dt}c_{Rt} = k_A c_{RNt} - k_P c_{Rt} \\
&\frac{d}{dt}c_{RP} = k_P c_{Rt} \qquad (21)
\end{aligned}
$$

Here, c_{RA} is the current concentration of R_A ribosomes; c_{T3} is that of T_3^T; c_{RT} is that of $R \cdot T_3^T$ complex; c_{RD} is that of $R \cdot T_3^D$ complex, c_{RNt} is the current concentration of $R{\cdot}Nt$ complex with non-accommodated aa-tRNA while c_{Rt} is that of $R{\cdot}t$ complex with accommodated aa-tRNA and c_{RP} is the concentration of $R{\cdot}P$ complex containing the extended peptide. All these concentrations are, of course, the functions of time. In addition to the accumulation of extended peptide one can also monitor (in the same experiment) the accumulation of GDP in $R \cdot T_3^D$ complex and in Tu·GDP dissociated from this complex [10,11].

Importantly, a GDP to GTP exchange factor EF-Ts that converts Tu·GDP back to Tu·GTP is excluded in such experiments to assure that GDP remains on EF-Tu after GTP hydrolysis for a sufficiently long time, about 3 min, to keep reacted EF-Tu inactive and unable to bind aa-tRNA for the whole duration of single cycle experiment [10,11].

Mean times of GTP hydrolysis and peptide bond formation

From this point on a further treatment of Supplementary Eq. (21) depends on whether a single cycle experiment is conducted with a large excess of R_A ribosomes over T_3^T or with a large excess of T_3^T over R_A ribosomes. The former setup is used in the experiments shown in Fig. 3a and 3b where the accumulation of both GDP and extended peptide is monitored in the same experiment (because in this set-up all GTP in T_3^T is hydrolyzed to GDP, providing a better GTP hydrolysis signal compared with the latter set-up). Importantly, in this setup the R_A concentration cannot reduce more that the total T_3^T initial concentration and can, therefore, be considered constant. We will now concentrate on the fate of T_3^T complex asking how fast its Tu·GTP moiety is converted to Tu·GDP and how fast its aa-tRNA moiety forms a new peptidyl-tRNA.

To find answers to these questions we treat Supplementary Eq. (21) in the following way. We first discard the first equation of the equation system (since we are not interested in R_A consumption) and then integrate both sides of the remaining equations (assuming constant c_{RA}) from the start of the reaction (time 0) to the time when all T_3^T hydrolyzed their GTP and their aa-tRNA either ended up in complex $R{\cdot}P$ with extended peptide or become discarded in the proofreading reaction. We denote this time as infinity, (∞), and normalize the result of integration to the initial concentration, $c_{T3}(0)$, of T_3^T. This leads to:

$$\begin{aligned}
-1 &= -k_1 c_{RA}\Theta_{T3} + q_1\Theta_{RT} \\
0 &= k_1 c_{RA}\Theta_{T3} - (q_1 + k_H)\Theta_{RT} \\
0 &= k_H\Theta_{RT} - k_{Tu\cdot GDP}\Theta_{RD} \\
1 &= k_{Tu\cdot GDP}\Theta_{RD} \\
0 &= k_{Tu\cdot GDP}\Theta_{RD} - (k_A + k_{AA-tRNA})\Theta_{RNt} \\
0 &= k_A\Theta_{RNt} - k_P\Theta_{Rt} \\
\frac{c_{RP}(\infty)}{c_{T3}(0)} &= k_P\Theta_{Rt}
\end{aligned} \tag{22}$$

Here, we formally introduced notations Θ_X for the integrals:

$$\Theta_X = \frac{1}{c_{T3}(0)}\int_0^\infty c_X(t)dt \tag{23}$$

We will see later that some of these integrals can be identified as mean life-times of the corresponding complexes. Appearance of several 1 and 0 on the left side in Supplementary Eq. (22) is because upon integration we obtain $(c_X(\infty) - c_X(0)) / c_{T3}(0)$ on the left side. Taking further into account that no intermediate complexes were present at time zero (i.e. $c_X(0) = 0$, except $c_{T3}(0)$) and that they all disappeared upon the experiment completion accumulated either as Tu·GDP or $R{\cdot}P$ (i.e. $c_X(\infty) = 0$, except $c_{RP}(\infty)$) one arrives at Supplementary Eq. (22). Solving it one obtains:

$$\Theta_{T3} = \frac{(1+q_1/k_H)}{k_1 c_{RA}} \qquad \Theta_{RT} = 1/k_H \qquad \Theta_{RD} = 1/k_{Tu\cdot GDP} \quad \text{and,} \quad \Theta_{RNt} = \frac{1}{k_A}\frac{1}{F} \qquad \Theta_{Rt} = \frac{1}{k_P}\frac{1}{F} \tag{24}$$

Here, F is a so called proofreading factor[12] also obtained from Supplementary Eq. (22) solution as:

$$F = (1 + k_{AA-tRNA} / k_A) = c_{T3}(0) / c_{RP}(\infty) \tag{25}$$

GTP hydrolysis

We note that when detecting GTP hydrolysis on EF-Tu in T_3 we actually measure the current concentration of produced GDP, $[GDP](t)$, both in complex $R \cdot T_3^D$ and in Tu·GDP dissociated from this complex, i.e., we measure:

$$[GDP](t) = c_{RD}(t) + [Tu \cdot GDP](t) \tag{26}$$

Further, it is easy to see from the law of matter conservation applied to EF-Tu that:

$$(c_{T3}(0) - [GDP](t)) = c_{T3}(t) + c_{RT}(t) \tag{27}$$

Dividing both parts of this relation by $c_{T3}(0)$ and integrating one obtains:

$$\int_0^\infty \frac{(c_{T3}(0) - [GDP](t))}{c_{T3}(0)} dt = \Theta_{T3} + \Theta_{RT} = \frac{(1+q_1/k_H)}{k_1 \cdot c_{RA}} + \frac{1}{k_H} \tag{28}$$

Here we used definitions of Θ_X provided in Supplementary Eq. (23) and their values from Supplementary Eq. (24). We will further see that Θ_{T3} and Θ_{RT} can be interpreted as mean life times of free T_3^T and of complex $R \cdot T_3^T$, respectively. Since the time course $[GDP](t)$ is obtained experimentally and $c_{T3}(0)$ is known, the numeric integration on the left side can be easily performed and the sum on the right side of Supplementary Eq. (28) recovered. Further, since all GTP initially in T_3^T is hydrolyzed to GDP in this experiment we have $c_{T3}(0) = [GDP](\infty)$. The integral on the left side in Supplementary Eq. (28) can be interpreted as a mean time of GTP hydrolysis, τ_{GTP}, and the equation re-written as:

$$\tau_{GTP} = \int_0^\infty \frac{([GDP](\infty) - [GDP](t))}{[GDP](\infty)} dt = \frac{1}{(k_{cat}/K_M)_{GTP} \cdot c_{RA}} + \frac{1}{k_{cat}^{GTP}} \tag{29}$$

Here:

$$(k_{cat}/K_M)_{GTP} = k_1/(1+q_1/k_H) = k_1 \cdot \{k_H/(k_H+q_1)\} \qquad k_{cat}^{GTP} = k_H \tag{30}$$

The meaning of k_{cat}/K_M notation

Parameters k_{cat}/K_M and k_{cat} defined by Supplementary Eq. (30) conforms with a general definition of k_{cat}/K_M as a kinetic efficiency of catalysis (catalysis of GTP hydrolysis in this case). Namely, k_{cat}/K_M is equal to the second order association rate constant k_1 for complex $R \cdot T_3^T$ formation multiplied by the probability, $k_H/(k_H+q_1)$, that the reaction will proceed to

the product, GDP, from this complex [9,12]. We note that Supplementary Eq. (29) holds also for more detailed kinetic schemes in which T_3 pre-binding and codon recognition steps are considered separately[8] and not lump together as here. However, the expressions for $(k_{cat}/K_M)_{GDP}$ and k_{cat}^{GTP} through the rate constants for such more detailed schemes become a bit more involved[8] and are not considered here. We note also that varying the concentration of R_A ribosomes, c_{RA} in this single cycle experiment one can extract both $(k_{cat}/K_M)_{GDP}$ and k_{cat}^{GTP} from variations in the meantime of GTP hydrolysis, τ_{GTP}.

Di-peptide formation

To see how one obtains mean time of extended peptide formation, τ_{EP}, called τ_{Dip} in the main text in experiments in Fig. 3b, we apply the law of matter conservation to aa-tRNA that was initially in T_3. It requires that:

$$c_{T3}(0) = c_{T3}(t) + c_{RT}(t) + c_{RD}(t) + c_{RNt}(t) + \left[aa - tRNA\right](t) + c_{Rt}(t) + c_{RP}(t) \tag{31}$$

To obtain the current concentration of aa-tRNA discarded at the proofreading step (during accommodation) we consider three last equations in Supplementary Eq. (21), sum up the last two and integrate both parts from time 0 to current time t. One obtains:

$$\left[aa - tRNA\right](t) = k_{AA-tRNA}\int_0^t c_{RNt}(T)dT$$

$$(c_{Rt}(t) + c_{RP}(t)) = k_A\int_0^t c_{RNt}(T)dT$$

From this it follows that:

$$\left[aa - tRNA\right](t) = (k_{AA-tRNA}/k_A)(c_{Rt}(t) + c_{RP}(t)) \tag{32}$$

Substituting this expression in Supplementary Eq. (31) and rearranging (recalling that $F = c_{T3}(0)/c_{RP}(\infty)$ in Supplementary Eq. (25)) one obtains after some algebra:

$$\frac{c_{RP}(\infty) - c_{RP}(t)}{c_{RP}(\infty)} = \frac{1}{c_{T3}(0)}(c_{T3}(t) + c_{RT}(t) + c_{RD}(t) + c_{RNt}(t)) + \frac{1}{F}\frac{c_{Rt}(t)}{c_{T3}(0)} \tag{33}$$

Integrating both parts of this relation one finds that:

$$\int_0^\infty \frac{c_{RP}(\infty) - c_{RP}(t)}{c_{RP}(\infty)}dt = \Theta_{T3} + \Theta_{RT} + \Theta_{RD} + \Theta_{RNt} + \frac{1}{F}\Theta_{Rt} \tag{34}$$

The integral on the left side in Supplementary Eq. (34) can be interpreted as a mean time τ_{EP} of peptide extension by one amino acid in single cycle experiment [9]. In experiments in Fig. 3B we start with 70S IC in place of R_A so that peptide extension results in dipeptide formation, and we will use τ_{Dip} instead of τ_{EP} here to conform with the main text notations. Using Θ_X values from Supplementary Eq. (24) plus $k_{\mathrm{cat}}/K_{\mathrm{M}}$ and k^{cat} definitions in Supplementary Eq. (30) one can re-write Supplementary Eq. (34) as:

$$\tau_{Dip} = \int_0^\infty \frac{c_{RP}(\infty) - c_{RP}(t)}{c_{RP}(\infty)}dt = \frac{1}{(k_{cat}/K_M)_{GTP}\cdot c_{RA}} + \frac{1}{k_{cat}^{GTP}} + \frac{1}{k_{Tu\cdot GDP}} + \frac{1}{k_A + k_{aa-tRNA}} + \frac{1}{k_P} \tag{35}$$

Since the time course $c_{RP}(t)$ is obtained experimentally, the numeric integration on the left can be easily performed and the sum of mean-times on the right side in Supplementary Eq. (35)

recovered. By varying the concentration of R_A ribosomes, c_{RA} , $(k_{cat}/K_M)_{GTP}$ can be extracted from τ_{Dip} variations [9].

Comparing Supplementary Eqs. (35) and (29) one concludes that subtracting τ_{GTP} from τ_{Dip} one obtains the sum $(1/k_{Tu\cdot GDP} + 1/(k_A + k_{aa\text{-}tRNA}) + 1/k_P)$ that can be interpreted as a sum of mean life-times of $R\cdot T_3^D$, $R{\cdot}Nt$ and $R{\cdot}t$ complexes (see Supplementary Fig. 4b). The first term describes a very fast $Tu\cdot GDP$ dissociation from $R\cdot T_3^D$ complex and can be neglected, so that subtracting τ_{GTP} from τ_{Dip} one obtain the sum $1/(k_A + k_{aa\text{-}tRNA}) + 1/k_P$ that gives the low bound for the sum $1/k_A + 1/k_P$ of mean times of aa-tRNA accommodation and peptidyl transfer[10,11].

Why do we fit the time courses of GTP hydrolysis or Dipeptide formation by a weighted sum of exponential functions?

We first note that when ribosome concentration is much higher than that of T_3 Supplementary Eq. (21) can be considered as a linear one (because the $k_1 c_{RA}(0)$ is approximately constant). It can, therefore, be readily solved by a variety of well- known methods (see Ref[13], for example). Importantly, the solution for time course of each concentration $c_X(t)$, including $c_{RP}(t)$ is a weighted sum of exponential functions of type $\exp(-\lambda_i t)$ plus, eventually a constant, while $(c_{RP}(\infty) - c_{RP}(t))/c_{RP}(\infty)$ is always a sum of weighted $\exp(-\lambda_i t)$ without a constant. It is then easy to see that fitting $(c_{RP}(\infty) - c_{RP}(t))/c_{RP}(\infty)$ by a weighted sum of exponential functions is just an alternative and more convenient way to carry out numerical integration in Supplementary Eq. (35). Namely:

$$\tau_{Dip} = \int_0^\infty \frac{(c_{RP}(\infty) - c_{RP}(t))}{c_{RP}(\infty)} dt = \int_0^\infty dt \frac{1}{\sum W_i} \sum W_i \exp(-\lambda_i t) = \frac{1}{\sum W_i} \sum \frac{W_i}{\lambda_i} \tag{36}$$

Here, the number of exponents is equal to the number of complexes in the scheme and λ_i-values are related (sometimes through rather involved algebra[13]) to the rate constants of the kinetic scheme in Supplementary Fig. 4b. Normally, many λ_i-values are much larger than others and their weights are small. That explains why one rarely fits the experimental di-peptide formation curve $c_{RT}(t)$ (denoted usually as $c_{Dip}(t)$) using more than 2-3 weighted exponentials. In practice, $c_{Dip}(t)$ curve is normally fitted using a “two-exponential” fit equation for a two-step reaction [5]:

$$c_{Dip}(t) = c_{Dip}(\infty)\left(1 - \frac{\lambda_1 e^{-\lambda_2 t} - \lambda_2 e^{-\lambda_1 t}}{\lambda_1 - \lambda_2}\right) \tag{37}$$

Another advantage of “exponential curve fitting” over direct numeric integration is that the fitting supplies errors of λ_i-parameters in Supplementary Eq. (36) from which the error in mean time τ_{Dip} can be extracted [11]. We note that in the case of GTP hydrolysis the single exponential fit:

$$c_{GDP}(t) = c_{GDP}(\infty)(1 - e^{-\lambda\cdot t}) \tag{38}$$

is normally used because the second term with k_H in Supplementary Eq. (28) is much smaller than the first term and can be neglected [9]. Importantly, this also implies that in the case of GTP hydrolysis λ-parameter is given by:

$$\lambda = (k_{cat} / K_M)_{GTP} \cdot c_{RA} \tag{39}$$

This further implies that $(k_{cat} / K_M)_{GTP}$ can be obtained as λ / c_{RA}.

Single cycle experiments for the case of large excess of T_3 over* R$_A$ *ribosomes.

We again consider kinetic scheme in Supplementary Fig. 4b, but assume a large excess of ternary complexes T_3 over R$_A$ ribosome as in experiments in Figure 3C. In this case we can assume T_3 concentration constant, exclude the second equation in Supplementary Eq. (21) (since we are not interested in free T_3 dynamics) and integrate both sides of the equations describing the dynamics of ribosome complexes from the start of the reaction (time 0) to the time when all R$_A$ ribosomes ended up in complex $R{\cdot}P$ (we denote this time as infinity, ∞). This time, we will, however, normalize the result of integration by the total concentration of ribosomes equal to $c_{RA}(0)$.

One obtains:

$$\begin{aligned}
-1 &= -k_1 c_{T3} \tau_{RA} + q_1 \tau_{RT} + k_{AA-tRNA} \tau_{RD} \\
0 &= k_1 c_{T3} \tau_{RA} - (q_1 + k_H) \tau_{RT} \\
0 &= k_H \tau_{RT} - k_{Tu \cdot GDP} \tau_{RD} \\
0 &= k_{Tu \cdot GDP} \tau_{RD} - (k_A + k_{aa-tRNA}) \tau_{RNt} \\
0 &= k_A \tau_{RNt} - k_P \tau_{Rt} \\
1 &= k_P \tau_{Rt}
\end{aligned} \tag{40}$$

Here, instead of Θ_X -notations of Supplementary Eq. (23), we formally introduced "mean-time" notations τ_X for the integrals:

$$\tau_X = \frac{1}{c_{RA}(0)} \int_0^\infty c_X(t) dt \tag{41}$$

Again, the appearance of several 1 and 0 on the left side in Supplementary Eq. (40) is because the integration produces $(c_X(\infty) - c_X(0)) / c_{RA}(0)$ there. Taking further into account the initial (i.e. $c_X(0) = 0$, except $c_{RA}(0)$) and final (i.e. $c_X(\infty) = 0$, except $c_{RP}(\infty) = c_{RA}(0)$) conditions one arrives at Supplementary Eq. (40). Solving it one obtains for mean life-times of different complexes:

$$\begin{aligned}
\tau_{RA} &= F \frac{(1 + q_1 / k_H)}{k_1 c_{T3}} \\
\tau_{RT} &= F / k_H \\
\tau_{RD} &= F / k_{Tu \cdot GDP}
\end{aligned}
\qquad \text{and,} \qquad
\begin{aligned}
\tau_{RNt} &= 1 / k_A \\
\tau_{Rt} &= 1 / k_P
\end{aligned} \tag{42}$$

Here, the proofreading factor $F = (1 + k_{AA-tRNA} / k_A)$ is the same as in Supplementary Eq. (25) except that the second equality in that equation is not applicable here.

From the law of matter conservation applied to the ribosomes it follows that:

$$c_{RA}(0) - c_{RP}(t) = c_{RA}(t) + c_{RT}(t) + c_{RD}(t) + c_{RNt}(t) + c_{Rt}(t)$$

Integrating both parts of this relation and dividing by the total ribosome concentration (equal to initial $c_{RA}(0)$) one obtains:

$$\tau_{EP} = \int_0^\infty \frac{(c_{RP}(\infty) - c_{RP}(t))}{c_{RP}(\infty)} dt = \tau_{RA} + \tau_{RT} + \tau_{RD} + \tau_{RNt} + \tau_{Rt} \tag{43}$$

Here, we took into account that all R_A ribosomes end up in $R{\cdot}P$ complex with extended peptide meaning that $c_{RA}(0) = c_{RP}(\infty)$. The integral on the left side of the above equation is the mean time τ_{EP} of extended peptide formation which we will further call τ_{Dip} to conform with the main text notations. Further, using expressions for mean times from Supplementary Eq. (42) plus $k_{\mathrm{cat}}/K_{\mathrm{M}}$ definition (Supplementary Eq. (30)), we re-write Supplementary Eq. (43) as:

$$\tau_{Dip} = \int_0^\infty \frac{(c_{RP}(\infty) - c_{RP}(t))}{c_{RP}(\infty)} dt = F\left\{\frac{1}{(k_{cat}/K_M)_{GTP}\left[T_3^T\right]} + \frac{1}{k_{cat}^{GTP}} + \frac{1}{k_{Tu\cdot GDP}}\right\} + \frac{1}{k_A} + \frac{1}{k_P} \tag{44}$$

Again, since the time course $c_{RP}(t)$ of extended peptide accumulation is obtained experimentally, the numeric integration on the left side can be easily performed and the sum of mean-times on the right side in Supplementary Eqs. (43) and (44) recovered.

It is instructive to compare the mean time of di-peptide formation in Supplementary Eq. (35) with that in Supplementary Eq. (44). In the former case (Supplementary Eq. (35)) the ribosomes are in excess over T_3^T and T_3^T goes through one and only one cycle of GTP hydrolysis spending sequentially time $1/\{(k_{cat}/K_M)_{GTP} c_{RA}(0)\}$ in the free state, time $1/k_H$ in complex $R \cdot T_3^T$ and time $1/k_{TuGDP}$ in complex $R \cdot T_3^D$ after which its aa-tRNA spend time $1/(k_{\mathrm{A}}+k_{\mathrm{aa\text{-}tRNA}})$ in complex $R{\cdot}Nt$ and time $1/k_P$ in complex $R{\cdot}t$ until it is finally participates in peptide bond formation. In the case of Supplementary Eq. (44) (here T_3^T is in excess over ribosomes) the ribosome R_A spends time $1/\left\{(k_{cat}/K_M)_{GTP}\left[T_3^T\right]\right\}$ as free R_A before T_3^T binding, time $1/k_H$ in complex $R \cdot T_3^T$ and time $1/k_{TuGDP}$ in complex $R \cdot T_3^D$ until it comes to complex $R{\cdot}Nt$ and spends time $1/(k_{\mathrm{A}}+k_{\mathrm{aa\text{-}tRNA}})$ there. The main differences between the two cases is that in the former case aa-tRNA discarded from $R{\cdot}Nt$ in the proofreading step cannot be re-used again, while in the latter case the ribosome R_A discarded in the same proofreading step has another chance to bind a new T_3 to go through the same complexes again and again (F-times on average) until it finally reaches $R{\cdot}t$ and spends time $1/k_P$ to make a peptide bond. This F-time cycling of I until its arriving to $R{\cdot}t$ complex explains why mean life times of complexes before complex $R{\cdot}t$ (see Supplementary Fig. 4b) are F-fold longer in Supplementary Eq. (44) compared with those in Supplementary Eq. (35).

Formal definition and explanation of mean times meaning

It is easy to see that we can interpret the ratio $c_X(t)/\ c_{RA}(0)$ in Supplementary Eq. (41) as the probably $P_X(t)$ to find the ribosome in complex X at time *t*, i.e. :

$$\tau_X = \int_0^{\infty} \frac{c_X(t)}{c_{Tot}} dt = \int_0^{\infty} P_X(t) dt$$

Here, total ribosome concentration c_{Tot} is the same as $c_{RA}(0)$. Further, the time integral over $P_X(t)$ have the meaning of time the ribosome spends (on average) in complex X during single cycle experiment. To see this, we cover the time axis from 0 of experiment start to time T of experiment duration by intervals Δt_j with centers at t_j. The length of these time intervals is selected so that probability $P_X(t)$ is nearly constant inside the time interval. This means that, on average, the ribosome spends time $\Delta t_j \times P_X(t_j)$ in complex X during time interval Δt_j. Now, we ask what time the ribosome spends in complex X from time zero to T. This time is obviously the sum of times $\Delta t_j \times P_X(t_j)$ it spends in X during each of time intervals Δt_j that cover the time axis from 0 to T, and this sum converges to an integral:

$$\tau_X(T) = \sum_j \Delta t_j P_X(t_j) = \int_0^T P_X(t) dt$$

Thus, $\tau_i(T)$ is the average time the ribosome spends in complex X during an experiment of duration T. Now, the time it spends in complex X tills the experiment is assuredly over will be:

$$\tau_X \equiv \tau_X(\infty) = \int_0^{\infty} P_X(t) dt$$

This time τ_X is often called a "mean (life) time" of complex X (on the reaction pathway).

Extraction of translocation time from two-or three-peptide bond formation experiment.

In the two- or three-peptide bond formation experiments one allows the ribosome to go through 2 or 3 elongation cycles depicted in Supplementary Fig. 4a and accumulate peptide extended by 2 or 3 amino acids (Figs. 3d, 3e, 3f). This is achieved by using mRNA with an appropriately positioned stop codon. In this work we use 70S initiation complex (70S IC) as an R_A post-translocation ribosome analogue so that the two amino acid peptide extension experiment analyzed below can be called a tri-peptide formation experiment. The analysis will demonstrate that the mean time of tri-peptide formation is the sum of mean times of two peptide extensions plus mean time of translocation reaction. This assertion is usually based on the analysis of a three step kinetic scheme depicted in Supplementary Fig. 4c [14].

We will, however, demonstrate the validity of this important assertion for the complete kinetic scheme of elongation cycle depicted in Supplementary Fig. 4a in which ribosome goes through two complete elongation cycles and stops because of R_A encountering a stop codon. The analysis below can be readily extended to any number of cycles. The dynamic of concentration of ribosome complexes for the kinetic scheme in Supplementary Fig. 4a that is allowed to go for two cycles under condition of a large T_3 and EF-G excess over ribosomes is governed by the following set of differential equations for the first and second cycles of peptide extension:

$$\frac{d}{dt}c_{RA}^{(1)} = -k_1 c_{T3} c_{RA}^{(1)} + q_1 c_{RT}^{(1)} + k_{AA-tRNA} c_{RD}^{(1)}$$
$$\frac{d}{dt}c_{RT}^{(1)} = k_1 c_{T3} c_{RA}^{(1)} - (q_1 + k_H) c_{RT}^{(1)}$$
$$\frac{d}{dt}c_{RD}^{(1)} = k_H c_{RT}^{(1)} - k_{Tu\cdot GDP} c_{RD}^{(1)}$$
$$\frac{d}{dt}c_{RNt}^{(1)} = k_{Tu\cdot GDP} c_{RD}^{(1)} - (k_A + k_{AA-tRNA}) c_{RNt}^{(1)}$$
$$\frac{d}{dt}c_{Rt}^{(1)} = k_A c_{RNt}^{(1)} - k_P c_{Rt}^{(1)}$$
$$\frac{d}{dt}c_{RP}^{(1)} = k_P c_{Rt}^{(1)} - k_G [G] c_{RP}^{(1)} + q_G c_{RPG}^{(1)}$$
$$\frac{d}{dt}c_{RPG}^{(1)} = k_G [G] c_{RP}^{(1)} - (q_G + k_T) c_{RPG}^{(1)}$$

and

$$\frac{d}{dt}c_{RA}^{(2)} = -k_1 c_{T3} c_{RA}^{(2)} + q_1 c_{RT}^{(2)} + k_{AA-tRNA} c_{RNt}^{(2)} + k_T c_{RPG}^{(1)}$$
$$\frac{d}{dt}c_{RT}^{(2)} = k_1 c_{T3} c_{RA}^{(2)} - (q_1 + k_H) c_{RT}^{(2)}$$
$$\frac{d}{dt}c_{RD}^{(2)} = k_H c_{RT}^{(2)} - k_{Tu\cdot GDP} c_{RD}^{(2)}$$
$$\frac{d}{dt}c_{RNt}^{(2)} = k_{Tu\cdot GDP} c_{RD}^{(2)} - (k_A + k_{AA-tRNA}) c_{RNt}^{(2)}$$
$$\frac{d}{dt}c_{Rt}^{(2)} = k_A c_{RNt}^{(2)} - k_P c_{Rt}^{(2)}$$
$$\frac{d}{dt}c_{RP}^{(2)} = k_P c_{Rt}^{(2)} - k_G [G] c_{RP}^{(2)} + q_G c_{RPG}^{(2)}$$
$$\frac{d}{dt}c_{RPG}^{(2)} = k_G [G] c_{RP}^{(2)} - (q_G + k_T) c_{RPG}^{(2)}$$
$$\frac{d}{dt}c_{R(UAA)}^{(3)} = k_T c_{RPG}^{(2)}$$

We first note that the time course of the accumulation of two-amino acids extended peptide is given by the sum $c_{RP}^{(2)}(t) + c_{RPG}^{(2)}(t) + c_{R(UAA)}^{(3)}(t)$ because we do not discriminate between pre- and post-translocated peptide of the same length in quench flow experiments. For brevity we introduce $c_{Trip}(t) = c_{RP}^{(2)}(t) + c_{RPG}^{(2)}(t) + c_{R(UAA)}^{(3)}(t)$ and call it a current tri-peptide concentration. We can, therefore, simplify the second cycle dynamics to:

$$\frac{d}{dt}c_{RA}^{(2)} = -k_1 c_{T3} c_{RA}^{(2)} + q_1 c_{RT}^{(2)} + k_{AA-tRNA} c_{RNt}^{(2)} + k_T c_{RPG}^{(1)}$$
$$\frac{d}{dt}c_{RT}^{(2)} = k_1 c_{T3} c_{RA}^{(2)} - (q_1 + k_H) c_{RT}^{(2)}$$
$$\frac{d}{dt}c_{RD}^{(2)} = k_H c_{RT}^{(2)} - k_{Tu\cdot GDP} c_{RD}^{(2)}$$
$$\frac{d}{dt}c_{RNt}^{(2)} = k_{Tu\cdot GDP} c_{RD}^{(2)} - (k_A + k_{AA-tRNA}) c_{RNt}^{(2)}$$
$$\frac{d}{dt}c_{Rt}^{(2)} = k_A c_{RNt}^{(2)} - k_P c_{Rt}^{(2)}$$
$$\frac{d}{dt}(c_{RP}^{(2)} + c_{RPG}^{(2)} + c_{R(UAA)}^{(3)}) = \frac{d}{dt}c_{Trip}(t) = k_P c_{Rt}^{(2)}$$

Next, we integrate the first and modified second cycle equations assuming that all ribosomes (70S IC in our case) went through the two cycles during experiment duration and ended up as ribosomes carrying tri-peptide in the P- and stop codon in the A-site of the *R(UAA)* ribosome. One obtains:

$$
\begin{aligned}
-1 &= -k_1 c_{T3}\tau_{RA}^{(1)} + q_1\tau_{RT}^{(1)} + k_{AA-tRNA}\tau_{RNt}^{(1)} \\
0 &= k_1 c_{T3}\tau_{RA}^{(1)} - (q_1 + k_H)\tau_{RT}^{(1)} \\
0 &= k_H\tau_{RT}^{(2)} - k_{Tu\cdot GDP}\tau_{RD}^{(2)} \\
0 &= k_{Tu\cdot GDP}\tau_{RD}^{(1)} - (k_A + k_{AA-tRNA})\tau_{RNt}^{(1)} \\
0 &= k_A\tau_{RNt}^{(1)} - k_P\tau_{Rt}^{(1)} \\
0 &= k_P\tau_{Rt}^{(1)} - k_G[G]\tau_{RP}^{(1)} + q_G\tau_{RPG}^{(1)} \\
0 &= k_P\tau_{Rt}^{(1)} - k_G[G]\tau_{RP}^{(1)} + q_G\tau_{RPG}^{(1)} \\
0 &= k_G[G]\tau_{RP}^{(1)} - (q_G + k_T)\tau_{RPG}^{(1)}
\end{aligned}
\qquad (45A)
$$

and

$$
\begin{aligned}
0 &= k_T\tau_{RPG}^{(1)} - k_1 c_{T3}\tau_{RA}^{(2)} + q_1\tau_{RT}^{(2)} \\
&+ k_{AA-tRNA}\tau_{RNt}^{(2)} \\
0 &= k_1 c_{T3}\tau_{RA}^{(2)} - (q_1 + k_H)\tau_{RT}^{(2)} \\
0 &= k_H\tau_{RT}^{(2)} - k_{Tu\cdot GDP}\tau_{RD}^{(2)} \\
0 &= k_{Tu\cdot GDP}\tau_{RD}^{(2)} - (k_A + k_{AA-tRNA})\tau_{RNt}^{(2)} \\
0 &= k_A\tau_{RNt}^{(2)} - k_P\tau_{Rt}^{(2)} \\
1 &= k_P\tau_{Rt}^{(2)}
\end{aligned}
\qquad (45B)
$$

Solving the above equation systems, one obtains:

$$
\begin{aligned}
\tau_{RA}^{(1)} &= F(1 + q_1 / k_H)/(k_1 c_{T3}) \\
\tau_{RT}^{(1)} &= F / k_H \\
\tau_{RD}^{(1)} &= F / k_{Tu\cdot GDP} \\
\tau_{RNt}^{(1)} &= 1 / k_A \\
\tau_{Rt}^{(1)} &= 1 / k_P \\
\tau_{RP}^{(1)} &= (1 + q_G / k_T)/(k_G[G]) \\
\tau_{RPG}^{(1)} &= 1 / k_T
\end{aligned}
\qquad (46A)
$$

and

$$
\begin{aligned}
\tau_{RA}^{(2)} &= F\frac{(1 + q_1 / k_H)}{k_1 c_{T3}} \\
\tau_{RT}^{(2)} &= F / k_H \\
\tau_{RD}^{(2)} &= F / k_{Tu\cdot GDP} \\
\tau_{RNt}^{(2)} &= 1 / k_A \\
\tau_{Rt}^{(2)} &= 1 / k_P
\end{aligned}
\qquad (46B)
$$

Further, applying the law of matter conservation to the ribosomes one gets:

$$
\begin{aligned}
c_{Tot} - c_{Trip}(t) &= (c_{RA}^{(1)}(t) + c_{RT}^{(1)}(t) + c_{RD}^{(1)}(t) + c_{RNt}^{(1)}(t) + c_{Rt}^{(1)}(t)) \\
&+ (c_{RP}^{(1)}(t) + c_{RPG}^{(1)}(t)) + (c_{RA}^{(2)}(t) + c_{RT}^{(2)}(t) + c_{RD}^{(2)}(t) + c_{RNt}^{(2)}(t) + c_{Rt}^{(2)}(t))
\end{aligned}
\qquad (47)
$$

The mean time of two-amino acid extended peptide (tri-peptide) formation τ_{Trip} is then:

$$
\begin{aligned}
\tau_{Trip} &= \int_0^\infty \frac{(c_{Tot} - c_{Trip}(t))}{c_{Tot}}dt = \int_0^\infty \frac{(c_{Trip}(\infty) - c_{Trip}(t))}{c_{Trip}(\infty)}dt \\
&= (\tau_{RA}^{(1)} + \tau_{RT}^{(1)} + \tau_{RD}^{(1)} + \tau_{RNt}^{(1)} + \tau_{Rt}^{(1)}) + (\tau_{RP}^{(1)} + \tau_{RPG}^{(1)}) + (\tau_{RA}^{(2)} + \tau_{RT}^{(2)} + \tau_{RD}^{(2)} + \tau_{RNt}^{(2)} + \tau_{Rt}^{(2)})
\end{aligned}
\qquad (48)
$$

Here, the left side is obtained from experimentally measured time course of tri-peptide accumulation. Comparing Supplementary Eqs (48) and (43) one concludes that on the right side the sum of mean times in the first and third brackets can be interpreted as mean time of dipeptide formation τ_{Dip} while the sum $(\tau_{RP}^{(1)} + \tau_{RPG}^{(1)})$ is the mean time for translocation:

$$
\tau_{Trans} = \tau_{RP}^{(1)} + \tau_{RPG}^{(1)} = \frac{(1 + q_G / k_T)}{k_G[G]} + \frac{1}{k_T} \qquad (49)
$$

The first term in τ_{Trans} to the right correspond to the mean time of EF-G binding to pre-translocation ribosome and the second term is the mean time of translocation by ribosome

bound EF-G [14]. This explains why the mean translocation time (at a given EF-G concentration) can be obtained from tri-peptide experiment by subtracting two mean times of di-peptide formation.

Similar analysis for the case of tetra-peptide shows that:

$$\tau_{Tetra} = \int_0^{\infty} \frac{(c_{Tetra}(\infty) - c_{Tetra}(t))}{c_{Tetra}(\infty)} dt = \tau_{Dip}^{(1)} + \tau_{Trans}^{(1)} + \tau_{Dip}^{(2)} + \tau_{Trans}^{(2)} + \tau_{Dip}^{(3)} \quad (50)$$

In is important to point out that mean time of di-peptide formation can be extracted directly from tri-peptide formation experiment excluding the necessity of a separate di-peptide experiment. This is because when monitoring the time course of tri-peptide accumulation by HPLC one also simultaneously monitors the time course of di-peptide [15]. In the case of di-peptide one actually monitors the sum, $c_{Dip}(t)$ of di-peptides in all ribosomal complexes that contain di-peptide, i.e. :

$$c_{Dip}(t) = c_{RP}^{(1)}(t) + c_{RPG}^{(1)}(t)) + c_{RA}^{(2)}(t) + c_{RT}^{(2)}(t) + c_{RD}^{(2)}(t) + c_{RNt}^{(2)}(t)$$

It is easy to see then that:

$$c_{Tot} - (c_{Dip}(t) + c_{Trip}(t)) = c_{RA}^{(1)}(t) + c_{RT}^{(1)}(t) + c_{RD}^{(1)}(t) + c_{RNt}^{(1)}(t) + c_{Rt}^{(1)}(t))$$

So that:

$$\int_0^{\infty} \frac{(c_{Tot} - (c_{Dip}(t) + c_{Trip}(t))}{c_{Tot}} dt = (\tau_{RA}^{(1)} + \tau_{RT}^{(1)} + \tau_{RD}^{(1)} + \tau_{RNt}^{(1)} + \tau_{Rt}^{(1)}) = \tau_{Dip} \quad (51)$$

Thus, we can extract τ_{Dip} from the time evolution of $c_{Dip}(t) + c_{Trip}(t)$ [15].

We note that tripeptide formation curve $c_{Trip}(t)$ is usually fitted using "three step kinetic scheme equation" [15]:

$$c_{Trip}(t) = c_{Trip}(\infty)\left\{1 + \frac{\lambda_2\lambda_3 e^{-\lambda_1 t}}{(\lambda_2 - \lambda_1)(\lambda_1 - \lambda_3)} + \frac{\lambda_1\lambda_3 e^{-\lambda_2 t}}{(\lambda_1 - \lambda_2)(\lambda_2 - \lambda_3)} + \frac{\lambda_1\lambda_2 e^{-\lambda_3 t}}{(\lambda_1 - \lambda_3)(\lambda_3 - \lambda_2)}\right\} \quad (52)$$

Here, $\lambda_1 = \lambda_3 = 1/\tau_{Dip}$ and $\lambda_2 = 1/\tau_{Trans}$ in "three step kinetic scheme" (Supplementary Fig. 4c).

Relation between "single cycle" and steady-state Michaelis-Menten kinetics.

It is often asked how the k_{cat}, K_M and k_{cat}/K_M parameters pertaining to the realm of steady-state Michaelis-Menten kinetics can be obtained from "single-cycle" pre-steady-state experiments. To see the connection between "single-cycle" and Michaelis-Menten kinetics we first notice that the net result of one elongation cycle in Supplementary Fig. 4a is the peptide extension by one amino-acid which can be also viewed as a one peptide bond addition cycle by a ribosome-enzyme. We can thus consider the kinetic scheme in Supplementary Fig. 4a as the scheme describing transitions of initially free enzyme R_A (which is ribosome R_A to the left) to its first bound state $R \cdot T_3^T$ and then through its different bound states to peptide bond addition and further back to a free enzyme R_A after translocation. The dynamics of the concentrations of free enzymes and six different bound

enzyme complexes in Supplementary Fig. 4a is governed by the following system of ordinary differential equations:

$$\begin{aligned}
\frac{d}{dt}c_{RA} &= -k_1\left[T_3^T\right]c_{RA} + q_1 c_{RT} + k_{AA-tRNA}c_{RNt} + k_T c_{RPG} \\
\frac{d}{dt}c_{RT} &= k_1\left[T_3^T\right]c_{RA} - (q_1 + k_H)c_{RT} \\
\frac{d}{dt}c_{RD} &= k_H c_{RT} - k_{Tu\cdot GDP}c_{RD} \\
\frac{d}{dt}c_{RNt} &= k_{Tu\cdot GDP}c_{RD} - (k_A + k_{AA-tRNA})c_{RNt} \\
\frac{d}{dt}c_{Rt} &= k_A c_{RNt} - k_P c_{Rt} \\
\frac{d}{dt}c_{RP} &= k_P c_{Rt} - k_G\left[G\right]c_{RP} + q_G c_{RPG} \\
\frac{d}{dt}c_{RPG} &= k_G\left[G\right]c_{RP} - (q_G + k_T)c_{RPG}
\end{aligned} \tag{53}$$

Assuming now that the concentrations of free enzyme and all enzyme bound complexes reached their steady states (alone with T_3^T and EF-G concentrations) and stopped changing, one can set all the derivatives on the left side of Supplementary Eq. (53) to zeros, move the term $k_T c_{RPG}$ in the first equation to the left and obtain steady state concentrations by solving the following system of algebraic equations:

$$\begin{aligned}
-k_T c_{RPG} &= -k_1\left[T_3^T\right]c_{RA} + q_1 c_{RT} + k_{AA-tRNA}c_{RNt} \\
0 &= k_1\left[T_3^T\right]c_{RA} - (q_1 + k_H)c_{RT} \\
0 &= k_H c_{RT} - k_{Tu\cdot GDP}c_{RD} \\
0 &= k_{Tu\cdot GDP}c_{RD} - (k_A + k_{AA-tRNA})c_{RNt} \\
0 &= k_A c_{RNt} - k_P c_{Rt} \\
0 &= k_P c_{Rt} - k_G\left[G\right]c_{RP} + q_G c_{RPG} \\
0 &= k_G\left[G\right]c_{RP} - (q_G + k_T)c_{RPG}
\end{aligned} \tag{54}$$

The easiest way to do this is first to introduce flow $j = k_P c_{Rt}$ which gives the number of peptide bond produced per unit volume per second. One can then solve Supplementary Eq. (54) to express all the steady state concentrations through this flow j as:

$$\begin{aligned}
c_{RA} &= jF\frac{(1+q_1/k_H)}{k_1\left[T_3^T\right]} \\
c_{RT} &= jF/k_H \\
c_{RD} &= jF/k_{Tu\cdot GDP}
\end{aligned}
\quad \text{and} \quad
\begin{aligned}
c_{RNt} &= j/k_A \\
c_{Rt} &= j/k_P \\
c_{RP} &= j\frac{(1+q_G/k_T)}{k_G\left[G\right]} \\
c_{RPG} &= j/k_T
\end{aligned} \tag{55}$$

Here F is given by the first relation in Supplementary Eq. (25). Comparing Supplementary Eqs. (55) and (46A), one finds that the steady state concentrations in Supplementary Eq. (55) are proportional to the mean life-times of the corresponding complex in Supplementary Eq. (46A) with the proportionality coefficient being flow j. This proportionality is not coincidental. Indeed, taking into account that $j = k_T c_{RPG}$ (see Supplementary Eq. (55)), it is easy to see that Supplementary Eq. (54) re-written as the equation for j-normalized steady state concentrations c_X/j coincides with Supplementary Eq. (45A) for mean times $\tau_X^{(1)}$ of corresponding complexes for the first cycle of the kinetic scheme in Supplementary Fig. 4a. The mathematical equivalence of single-cycle equations for τ_X and steady state equations for c_X/j is quite general and can be demonstrated for many kinetic schemes that can operate both in single-cycle and multi-cycle modes.

Further, to obtain the Michaelis-Menten description from the steady–state description of the multi-cycling mode we just sum up the steady-state concentrations of all ribosome complexes in Supplementary Fig. 4a plus that of R_A. Such a sum must be equal to the total ribosome (enzyme) concentration c_{Tot}. In addition, we will take into account that j is the number of peptide bonds per second per unit volume produced by c_{Tot} ribosomes in the same unit volume, so that the cycle time τ_{cyc} that one ribosome spends on average to extent peptide by one amino-acid (mean elongation cycle time) is c_{Tot} / j . Using this and steady-state concentrations from Supplementary Eq. (55) one obtains:

$$\tau_{cyc} = c_{Tot} / j = c_{RA} / j + c_{RT} / j + c_{RD} / j + c_{RNt} / j + c_{Rt} / j + c_{RP} / j + c_{RPG} / j =$$
$$= F\frac{(1+q_1/k_H)}{k_1\left[T_3^T\right]} + \left\{\frac{F}{k_H} + \frac{F}{k_{Tu\cdot GDP}} + \frac{1}{k_A} + \frac{1}{k_P} + \frac{(1+q_G/k_T)}{k_G\left[G\right]} + \frac{1}{k_T}\right\} \quad (56)$$

One can then interpret each term in the sum in Supplementary Eq. (56) as the mean time the ribosome spends in the corresponding complex (see Supplementary Fig. 4a) during one elongation cycler while it goes around it. Furthermore, Supplementary Eq. (56) relates the mean elongation cycle time of ribosome τ_{cyc} with free T_3^T and EF-G concentrations and corresponds to Eadie–Hofstee formulation of Michaelis–Menten equation of enzyme kinetics. Namely, Eadie–Hofstee relation is:

$$\tau_{cyc} = \frac{\left[E_{Tot}\right]}{j} = \frac{1}{(k_{cat}/K_M)\left[S\right]} + \frac{1}{k_{cat}} \quad (57)$$

Comparing Supplementary Eqs (57) and (56) under assumption of very high EF-G concentration (so that G-dependent term in Supplementary Eq. (56) can be neglected) one can identify:

$$\frac{k_{cat}}{K_M} = \frac{1}{F}\frac{k_1}{(1+q_1/k_H)} \;;\; \left[S\right] = \left[T_3^T\right]; \;\; \frac{1}{k_{cat}} = \frac{F}{k_H} + \frac{F}{k_{Tu\cdot GDP}} + \frac{1}{k_A} + \frac{1}{k_P} + \frac{1}{k_T}$$

Supplementary Note 3: Termination phase of translation cycle and termination kinetics

The termination phase of translation cycle is depicted in Supplementary Fig. 14a. Here, terminating ribosome (R) with stop codon in the A-site binds release factor (F) with rate constant k_1 forming release factor pre-bound complex $R{\cdot}F$ from which F can either dissociate with rate constant q_1 or recognize stop codon with rate constant k_r forming complex $R{\cdot}F_r$. Complex $R{\cdot}F_r$ can either return back to $R{\cdot}F$ with date constant q_r or proceed forward with rate k_c to complex $R{\cdot}F_c$ in which release factor F undergone a conformation change that positions its GGQ loop into the PTC of the ribosome. This leads to the ester bond hydrolysis in peptidyl-tRNA with rate constant k_H and peptide release[16]. THB (T) can bind to R forming complex $R{\cdot}T$ and it can bind to $R{\cdot}F$ forming complex $R{\cdot}T{\cdot}F$. These THB binding reactions have rate constants depicted in Supplementary Fig. 14a.

The kinetics of concentrations of different complexes in Supplementary Fig. 14a are described by the follows set of differential equations:

$$
\begin{aligned}
&\frac{d}{dt}c_R = -(k_1[F]+k_2[T])c_R + q_1c_{RF} + q_2c_{RT} \\
&\frac{d}{dt}c_{RF} = k_1[F]c_R - (q_1 + k_4[T] + k_r)c_{RF} + q_4c_{RTF} + q_rc_{RFr} \\
&\frac{d}{dt}c_{RT} = k_2[T]c_R - (q_2 + k_3[F])c_{RT} + q_3c_{RTF} \\
&\frac{d}{dt}c_{RTF} = k_4[T]c_{RF} + k_3[F]c_{RT} - (q_3 + q_4)c_{RTF} \\
&\frac{d}{dt}c_{RFr} = k_rc_{RF} - (q_r + k_c)c_{RFr} \\
&\frac{d}{dt}c_{RFC} = k_cc_{RFr} - k_Hc_{RFC} \\
&\frac{d}{dt}c_{RH} = k_Hc_{RFC}
\end{aligned}
\tag{58}
$$

Here, we introduced the following notation for the ribosome complexes: $RF = R{\cdot}F$; $RT = R{\cdot}T$; $RTF = R{\cdot}T{\cdot}F$; $RFr = R{\cdot}Fr$; $RFC = R{\cdot}Fc$.

Mean-times of different complexes, τ_X are defined as in Supplementary Eq. (41):

$$\tau_X = \frac{1}{c_{tot}}\int_0^\infty c_X(t)dt \tag{59}$$

Here c_{tot} is the total ribosome concentration in different complexes equal to the initial concentration of terminating ribosomes R. The concentrations of free THB, $[T]$, and that of free RF2, $[F]$, are in a large excess over total ribosome concentration and remain approximately constant during the reaction progress. We can, therefore, integrate equation system Eq. (58) from time zero of experiment start to some long time denoted (∞) when all peptides are released (i.e., "hydrolyzed off" peptidyl-tRNA) assuming $[T]$ and $[F]$ constant. The integration results in:

$$
\begin{aligned}
&\frac{c_R(\infty)-c_R(0)}{c_{Tot}} = -(k_1[F]+k_2[T])\tau_R + q_1\tau_{RF} + q_2\tau_{RT} \\
&\frac{c_{RF}(\infty)-c_{RF}(0)}{c_{Tot}} = k_1[F]\tau_R - (q_1 + k_4[T] + k_r)\tau_{RF} + q_4\tau_{RTF} + q_r\tau_{RFr} \\
&\frac{c_{RT}(\infty)-c_{RT}(0)}{c_{Tot}} = k_2[T]\tau_R - (q_2 + k_3[F])\tau_{RT} + q_3\tau_{RTF} \\
&\frac{c_{RTF}(\infty)-c_{RTF}(0)}{c_{Tot}} = k_4[T]\tau_{RF} + k_3[F]\tau_{RT} - (q_3+q_4)\tau_{RTF} \\
&\frac{c_{RFr}(\infty)-c_{RFr}(0)}{c_{Tot}} = k_r\tau_{RF} - (q_r + k_c)\tau_{RFr} \\
&\frac{c_{RFC}(\infty)-c_{RFC}(0)}{c_{Tot}} = k_c\tau_{RFr} - k_H\tau_{RFC} \\
&\frac{c_{RHF}(\infty)-c_{RHF}(0)}{c_{Tot}} = k_H\tau_{RFC}
\end{aligned}
\qquad (60)
$$

Further, the matter conservation law applied to free terminating ribosomes and ribosomes in different complexes in Supplementary Fig. 14a requires that:

$$
c_R(t) + c_{RF}(t) + c_{RT}(t) + c_{RTF}(t) + c_{RFr}(t) + c_{RFC}(t) + c_{RFH}(t) = c_{Tot} \qquad (61)
$$

We define the mean-time, τ_P, of peptide release (from the P-site tRNA) as integral:

$$
\tau_P = \int_0^\infty \frac{c_{tot} - c_{RFH}(t)}{c_{tot}} dt \qquad (62)
$$

Taking into account the definition of mean-times in Supplementary Eq. (59) one finds that τ_P is the sum:

$$
\tau_P = \int_0^\infty \frac{c_{RFH}(\infty) - c_{RFH}(t)}{c_{RFH}(\infty)} dt = \tau_R + \tau_{RF} + \tau_{RT} + \tau_{RTF} + \tau_{RFr} + \tau_{RFC} \qquad (63)
$$

Here, we used that all terminated ribosomes released their peptides at the end of the experiment, so that $c_{tot} = c_{RFH}(\infty)$ in Supplementary Eq. (62).

Mean time of peptide release in the absence of THB

A single cycle experiment on peptide release starts with mixing terminating ribosomes R with release factor F. When THB is absent ([*T*] =0) it is obvious that the complexes *R·T* and *R·T·F* in Supplementary Fig. 14a are not populated, and their mean-times are zero. The kinetic scheme in Supplementary Fig. 14a reduces therefore to that in Supplementary Fig. 14b. For this scheme the equation system Eq. (60) reduces to:

$$
\begin{aligned}
&-1 = -k_1[F]\tau_R + q_1\tau_{RF} \\
&0 = k_1[F]\tau_R - (q_1 + k_r)\tau_{RF} + q_r\tau_{RFr} \\
&0 = k_r\tau_{RF} - (q_r + k_c)\tau_{RFr} \\
&0 = k_c\tau_{RFr} - k_H\tau_{RFC} \\
&1 = k_H\tau_{RFC}
\end{aligned}
\qquad (64)
$$

Here zeros and 1s on the right side come from initial and final conditions. Solving Supplementary Eq. (64) one obtains for the mean times:

$$\tau_R = \frac{1+(q_1/k_r)(1+q_r/k_c)}{k_1[F]}$$
$$\tau_{RF} = (1+q_r/k_c)/k_r \tag{65}$$
$$\tau_{RFr} = 1/k_c$$
$$\tau_{RFC} = 1/k_H$$

The meantime, τ_P of peptide release is then the sum of all mean times in Supplementary Eq. (65), which we compact to:

$$\tau_P = \frac{1}{(k_{cat}/K_M)_P[F]} + \frac{1}{k_{cat}} \tag{66}$$

Here k_{cat}/K_M and k_{cat} of the release reaction described by Supplementary Fig. 14b are:

$$(k_{cat}/K_M)_P = k_1/\{1+(q_1/k_r)(1+q_r/k_c)\}$$
$$k_{cat} = 1/\left\{\frac{(1+q_r/k_c)}{k_r} + \frac{1}{k_c} + \frac{1}{k_H}\right\} \tag{67}$$

Supplementary Eq. (66) shows that the dependence of mean-time τ_P of peptide release on release factor concentration [F] obeys Eadie–Hofstee formulation of standard Michaelis–Menten kinetics so that the standard k_{cat}/K_M and k_{cat} parameters can be extracted from [F] titration experiments as shown in Fig. 4A. We also note that the first term in Supplementary Eq. (66) is the mean life-time of release-factor-free terminating ribosome while the second term, $1/k_{cat}$, is the sum of mean life-times of release factor-bound terminating ribosomes (compare Supplementary Eqs. (65) and (67)).

Mean-time of peptide release in THB presence when THB does not dissociate from terminating ribosome

In the release experiments conducted in THB presence, THB was first pre-incubated with terminating ribosomes before release factor *F* addition. In this case all terminating ribosomes (*R*) are initially THB bound in complex *R·T*.

Let us first consider a kinetic scheme of release reaction in which THB, once bound to *R*, remains bound all the way through the reaction until peptide is released. The kinetic scheme describing this situation is depicted in Supplementary Fig. 14c. This scheme is formally identical to that in Supplementary Fig. 14b except for different names for complexes and rate constants. The "mean-time treatment" of this scheme is, therefore, identical to that of scheme in Supplementary Fig. 14b. One gets, therefore, for the mean time of peptide release, τ_P^{THB} under persistent THB presence:

$$\tau_P^{THB} = \frac{1}{(k_{cat}/K_M)_P^{THB}[F]} + \frac{1}{k_{cat}^{THB}} \tag{68}$$

The expressions for k_{cat}/K_M and k_{cat} parameters are formally identical to those in Supplementary Eq. (67) except for different rate constant notations:

$$(k_{cat}/K_M)_P^{THB} = k_3/(1+(q_3/k_r^{THB})(1+q_r^{THB}/k_c^{THB}))$$
$$k_{cat}^{THB} = 1/\left\{\frac{(1+q_r^{THB}/k_c^{THB})}{k_r^{THB}} + \frac{1}{k_c^{THB}} + \frac{1}{k_H^{THB}}\right\} \tag{69}$$

From structural consideration it is reasonable to assume that THB presence on the terminating ribosome will severely inhibit the codon recognition by release factor, i.e., that rate constant k_r^{THB} will be greatly reduced and q_r^{THB} greatly increased due to THB presence. We note that termination scheme in Supplementary Fig. 14a correspond to the extreme situation when $k_r^{THB} = 0$ in THB presence, so that THB has to dissociate from the ribosome for the peptide release to occur. Assuming that only k_r^{THB} and q_r^{THB} are affected by THB in Supplementary Fig. 14c and all other rate constants are as in Supplementary Fig. 14b we can use Supplementary Eq. (69) to obtain the following expressions for the inverses of k_{cat}/K_M and k_{cat} parameters:

$$\begin{aligned} 1/(k_{cat}/K_M)_P^{THB} &= \frac{1}{k_1}\left(1+q_1\frac{(1+q_r^{THB}/k_c)}{k_r^{THB}}\right) \\ 1/k_{cat}^{THB} &= \frac{(1+q_r^{THB}/k_c)}{k_r^{THB}} + \frac{1}{k_c} + \frac{1}{k_H} \end{aligned} \qquad (70)$$

Those should be compared with the corresponding expressions valid in THB absence:

$$\begin{aligned} 1/(k_{cat}/K_M)_P &= \frac{1}{k_1}\left(1+q_1\frac{(1+q_r/k_c)}{k_r}\right) \\ 1/k_{cat} &= \frac{(1+q_r/k_c)}{k_r} + \frac{1}{k_c} + \frac{1}{k_H} \end{aligned} \qquad (71)$$

Our experiments in Fig. 4 show that $1/k_{cat}$ increased from 0.17 s in THB absence to 45 s in its presence implying that $(1+q_r^{THB}/k_c)/k_r^{THB}$ is about 45 s too (compare expressions for $1/k_{cat}$ in Supplementary Eqs (70) and (71)). Further, we observed an about 4000-fold reduction in k_{cat}/K_M of the release reaction upon THB addition that implies a minimum 4000-fold higher value of $(1+q_r^{THB}/k_c)/k_r^{THB}$ than $(1+q_r/k_c)/k_r$ (compare expressions for $1/(k_{cat}/K_M)$ in Supplementary Eqs (70) and (71)). It then follows that $(1+q_r/k_c)/k_r$ should be less than 10 ms (since $(1+q_r^{THB}/k_c)/k_r^{THB}$ is 45 s in THB presence), implying a very fast rate k_r of codon recognition in $R{\cdot}F$ complex by the pre-bound release factor. Moreover, this 4,000-fold k_{cat}/K_M reduction requires the second term inside brackets in the first relation in Supplementary Eq. (70) to be much larger than 1, implying, in turn, that to a very good approximation:

$$1/(k_{cat}/K_M)_P^{THB} \approx K_1\frac{(1+q_r^{THB}/k_c)}{k_r^{THB}} \qquad (72)$$

From this also follows that K_M value of 13 μM measured in THB presence corresponds to the equilibrium dissociation constant $K_1=q_1/k_1$ of the release factor pre-binding step.

We can conclude therefore that our experimental results can be formally explained by the scenario depicted in Supplementary Fig. 14c in which the peptide release occurs on THB bound terminating ribosomes. This scenario explains also why the increase of THB concentration had no effect on the kinetics of peptide release in experiments in Supplementary Fig. 13b. This is because terminating ribosomes and all complexes on the pathway to peptide release already contain THB (see Supplementary Fig. 14c).

Mean-time of peptide release when THB dissociation from ribosome is required for the release

Let us now assume that THB and RF1/2 cannot pre-binding to terminating ribosome R simultaneously so that $R{\cdot}T{\cdot}F$ complex cannot be reached and THB dissociation from $R{\cdot}T$ complex is required for the release factor recognition of stop codon and peptide release. This reduces the scheme in Supplementary Fig. 14a to the scheme in Supplementary Fig. 14d. The equation set in Supplementary Eq. (60) reduces then to:

$$\begin{aligned}
0 &= -(k_1[F]+k_2[T])\tau_R + q_1\tau_{RF} + q_2\tau_{RT} \\
0 &= k_1[F]\tau_R - (q_1+k_r)\tau_{RF} + q_r\tau_{RFr} \\
-1 &= k_2[T]\tau_R - q_2\tau_{RT} \\
0 &= k_r\tau_{RF} - (q_r+k_c)\tau_{RFr} \\
0 &= k_c\tau_{RFr} - k_H\tau_{RFC} \\
1 &= k_H\tau_{RFC}
\end{aligned} \qquad (73)$$

Solving this equation, one obtains for mean time of $R{\cdot}T$ complex

$$\tau_{RT} = \frac{1}{q_2}\left[1 + k_2[T]\frac{1+(q_1/k_r)(1+q_r/k_c))}{k_1[F]}\right] = \frac{1}{q_2}\left[1 + \frac{k_2[T]}{(k_{cat}/K_M)_P[F]}\right]$$

The expressions for mean times τ_{RFC}, τ_{RFr}, τ_{RF} and τ_R are the same as in Supplementary Eq. (65). Summing up the relevant mean-times one obtains for the mean time of peptide release:

$$\tau_P = \frac{1+[T]/K_2}{(k_{cat}/K_M)_P[F]} + \frac{1}{k_{cat}} + \frac{1}{q_2} \qquad (74)$$

Here $K_2=q_2/k_2$ is the equilibrium dissociation constant for THB binding to terminating ribosome R and q_2 is the rate constant of THB dissociation from R. It is easy to see that effective k_{cat} and k_{cat}/K_M parameters obtained from $[F]$ titration experiments in THB presence are given by:

$$\begin{aligned}
(k_{cat}/K_M)_P^{eff} &= (k_{cat}/K_M)_P \frac{K_2}{K_2+[T]} \\
k_{cat}^{eff} &= 1/(1/q_2 + 1/k_{cat})
\end{aligned} \qquad (75)$$

The peptide release scenario described by Supplementary Fig. 14d explains, therefore, a drastic decrease in effective k_{cat} (due to a long time $1/q_2$ of THB dissociation) and a drastic decrease in effective k_{cat}/K_M (due to small K_2 for THB binding) in THB presence (see Supplementary Eq. (75)). However, Supplementary Eq. (74) predicts a strong τ_P increase with increasing THB concentration at a fixed release factor concentration not observed experimentally (see Supplementary Fig. 13b). Thus, the termination scheme in Supplementary Fig. 14d in which $R{\cdot}T{\cdot}F$ complex cannot be formed fails to explain our experimental results in Supplementary Fig. 13b.

Let us now consider the termination scheme depicted in Supplementary Fig. 14a in which the release can also proceed through the formation of $R{\cdot}T{\cdot}F$ complex. Considering that all terminating ribosomes R, are initially THB bound, i.e., are in complex $R{\cdot}T$, the equation system in Supplementary Eq. (60) becomes:

$$
\begin{aligned}
&0=-(k_1[F]+k_2[T])\tau_R+q_1\tau_{RF}+q_2\tau_{RT}\\
&0=k_1[F]\tau_R-(q_1+k_4[T]+k_r)\tau_{RF}+q_4\tau_{RTF}+q_r\tau_{RFC}\\
&-1=k_2[T]\tau_R-(q_2+k_3[F])\tau_{RT}+q_3\tau_{RTF}\\
&0=k_4[T]\tau_{RF}+k_3[F]\tau_{RT}-(q_3+q_4)\tau_{RTF}\\
&0=k_r\tau_{RF}-(q_r+k_c)\tau_{RFr}\\
&0=k_c\tau_{RFr}-k_H\tau_{RFC}\\
&1=k_H\tau_{RFC}
\end{aligned}
\tag{76}
$$

Its solution for mean-times τ_{RFC}, τ_{RFr} and τ_{RF} are the same as in Supplementary Eq. (65). Introducing an unknown parameter α as $\alpha=k_1[F]\tau_R-q_1\tau_{RF}$ one obtains from first four equation in Supplementary Eq. (76):

$$
\begin{aligned}
&q_2\tau_{RT}-k_2[T]\tau_R=\alpha\\
&k_1[F]\tau_R-q_1\tau_{RF}=\alpha\\
&1-\alpha=-k_4[T]\tau_{RF}+q_4\tau_{RTF}\\
&1-\alpha=k_3[F]\tau_{RT}-q_3\tau_{RTF}
\end{aligned}
$$

This allows one to express three remaining mean times through α and τ_{RF} as:

$$
\begin{aligned}
&\tau_R=\frac{1}{k_1[F]}(\alpha+q_1\tau_{RF})\\
&\tau_{RT}=\frac{1}{q_2}(\alpha+k_2[T]\tau_R)=\frac{\alpha}{q_2}+\frac{k_2[T]}{k_1[F]}\frac{1}{q_2}(\alpha+q_1\tau_{RF})\\
&\tau_{RTF}=\frac{1}{q_4}(1-\alpha+k_4[T]\tau_{RF})=\frac{1-\alpha}{q_4}+\frac{[T]}{K_4}\tau_{RF}\\
&\tau_{RT}=\frac{1}{k_3[F]}(1-\alpha+q_3\tau_{RTF})=\frac{K_3}{[F]}(\frac{1-\alpha}{q_3}+\tau_{RTF})=\frac{K_3}{[F]}(\frac{1-\alpha}{q_3}+\frac{1-\alpha}{q_4}+\frac{[T]}{K_4}\tau_{RF})
\end{aligned}
\tag{77}
$$

Here, $K_i=q_i/k_i$ (i=1, 2, 3 or 4) are equilibrium dissociation constants. Summing up all six mean times and re-arranging terms one obtains:

$$
\tau_P=\frac{1}{k_{cat}}+\frac{1}{k_1[F]}(q_1\tau_{RF}+\alpha)+\frac{K_3}{[F]}\frac{1-\alpha}{q_3}+(1+\frac{K_3}{[F]})\frac{1-\alpha}{q_4}+(1+\frac{K_3}{[F]})\frac{[T]}{K_4}\tau_{RF}
\tag{78}
$$

Here, k_{cat} is defined by Supplementary Eq. (67). Parameter alpha is then recovered from two alternative expressions for τ_{RT} in Supplementary Eq. (77):

$$
\tau_{RT}=\frac{\alpha}{q_2}+\frac{[T]}{K_2}\frac{\alpha}{k_1[F]}+\frac{[T]}{K_2}\frac{K_1}{[F]}\tau_{RF}=\frac{1-\alpha}{k_3[F]}+\frac{K_3}{[F]}\frac{1-\alpha}{q_4}+\frac{K_3}{[F]}\frac{[T]}{K_4}\tau_{RF}
$$

Taking into account that detailed balance requires $K_1/K_2=K_3/K_4$ one obtains for α:

$$
\alpha=\frac{K_3}{[F]}(\frac{1}{q_3}+\frac{1}{q_4})/\left\{\frac{1}{q_2}(1+\frac{k_2[T]}{k_1[F]})+\frac{K_3}{[F]}(\frac{1}{q_3}+\frac{1}{q_4})\right\}
\tag{79}
$$

Also:

$$1-\alpha=\frac{1}{q_2}(1+\frac{k_2[T]}{k_1[F]})/\left\{\frac{1}{q_2}(1+\frac{k_2[T]}{k_1[F]})+\frac{K_3}{[F]}(\frac{1}{q_3}+\frac{1}{q_4})\right\} \tag{80}$$

Here, we can safely assume that release factor F dissociates much faster that THB from $R{\cdot}T{\cdot}F$ complex meaning that $q_3 >> q_4$. Further, at high release factor concentration we can also safely assume that release factor binds to terminating ribosome R much faster than THB meaning that $k_2[T] << k_1[F]$. Using these reasonable assumptions, we can neglect terms containing $1/q_3$ in comparison with terms containing $1/q_4$ and simplify Supplementary Eq. (80) to:

$$1-\alpha\approx 1/\left\{1+\frac{K_3}{[F]}\frac{q_2}{q_4}\right\} \tag{81}$$

Further, in Supplementary Eq. (78) the second and third terms are much smaller in comparison with other terms and can be neglected. Using Supplementary Eq. (81) and expression for τ_{RF} (Supplementary Eq. (65)) Supplementary Eq. (78) for τ_P is reduced to:

$$\tau_P=\frac{1}{k_{cat}}+(1+\frac{K_3}{[F]})\frac{1}{q_4}/\left\{1+\frac{K_3}{[F]}\frac{q_2}{q_4}\right\}+(1+\frac{K_3}{[F]})\frac{[T]}{K_4}\frac{(1+q_r/k_c)}{k_r} \tag{82}$$

Let us assume now that release factor pre-binding to $R{\cdot}T$ destabilizes THB binding in $R{\cdot}T{\cdot}F$ complex, so that $K_4 >> K_2$ and $q_4 >> q_2$. Under these assumptions Supplementary Eq. (82) simplifies further to:

$$\tau_P\approx\frac{1}{k_{cat}}+\frac{1}{q_4}(1+\frac{k_4[T]}{k_r}(1+q_r/k_c))+\frac{K_3}{[F]}\frac{1}{q_4}(1+\frac{k_4[T]}{k_r}(1+q_r/k_c)) \tag{83}$$

It is also reasonable to assume that in a studied range of THB concentrations THB bind $R{\cdot}F$ complex much more slowly than $R{\cdot}F$ releases peptide with rate k_{cat}. This implies that:

$$\frac{k_4[T]}{k_r}(1+q_r/k_c)<\frac{k_4[T]}{k_{cat}}<<1$$

This further implies that in THB presence:

$$\tau_P\approx\frac{1}{k_{cat}}+\frac{1}{q_4}+\frac{K_3}{[F]}\frac{1}{q_4} \tag{84}$$

Therefore, the effective values of k_{cat} and k_{cat}/K_M for Scheme R1 are:

$$\begin{aligned}(k_{cat}/K_M)_P^{eff}&=q_4/K_3\\ k_{cat}^{eff}&=1/(1/q_4+1/k_{cat})\end{aligned} \tag{85}$$

Supplementary Eq. (85) shows that under assumption that the pre-binding of release factor in $R{\cdot}T{\cdot}F$ complex greatly destabilizes THB binding to it, Supplementary Fig. 14a can explain both great decrease in k_{cat} and k_{cat}/K_M as well as the absence of their dependence on THB concentration. Hence, kinetic scheme of termination in Supplementary Fig. 14a can also explain all experimental results in Fig. 4.

We note also that if the release factor pre-binding in $R{\cdot}T{\cdot}F$ complex had no effect on the rate constant of THB dissociation, i.e., if $q_4 = q_2$ then Supplementary Eq. (82) transforms into:

$$\tau_P \approx \frac{1}{k_{cat}} + \frac{1}{q_4}(1+\frac{k_4[T]}{k_r}(1+q_r/k_c)) + \frac{K_1}{[F]}\frac{[T]}{K_2}\frac{(1+q_r/k_c)}{k_r}$$
$$\approx \frac{1}{k_{cat}} + \frac{1}{q_4} + \frac{1}{[F]}\left\{\frac{K_1(1+q_r/k_c)}{k_r}\right\}\frac{[T]}{K_2} \qquad (86)$$

It shows that k_{cat}^{eff} is the same as in Supplementary Eq. (84) while k_{cat}/K_M value is expected to vary with THB concentration in a way similar to that deduced for kinetic scheme of termination in Supplementary Fig. 14d (see Supplementary Eq. (75)) that is incompatible with experimental data in Supplementary Fig. 13b.

Supplementary References

1 Antoun, A., Pavlov, M. Y., Lovmar, M. & Ehrenberg, M. How initiation factors tune the rate of initiation of protein synthesis in bacteria. *EMBO J* **25**, 2539-2550 (2006).

2 Simonetti, A. *et al.* Structure of the 30S translation initiation complex. *Nature* **455**, 416-420 (2008).

3 Antoun, A., Pavlov, M. Y., Andersson, K., Tenson, T. & Ehrenberg, M. The roles of initiation factor 2 and guanosine triphosphate in initiation of protein synthesis. *EMBO J* **22**, 5593-5601 (2003).

4 Balakin, A. G., Skripkin, E. A., Shatsky, I. N. & Bogdanov, A. A. Unusual ribosome binding properties of mRNA encoding bacteriophage lambda repressor. *Nucleic Acids Res* **20**, 563-571, doi:10.1093/nar/20.3.563 (1992).

5 Fersht, A. *Enzyme structure and mechanism.* 1977 edn, 371 (W.H. Freeman and Company, 1977).

6 Parajuli, N. P., Mandava, C. S., Pavlov, M. Y. & Sanyal, S. Mechanistic insights into translation inhibition by aminoglycoside antibiotic arbekacin. *Nucleic Acids Res* **49**, 6880-6892, doi:10.1093/nar/gkab495 (2021).

7 Pape, T., Wintermeyer, W. & Rodnina, M. V. Complete kinetic mechanism of elongation factor Tu-dependent binding of aminoacyl-tRNA to the A site of the E. coli ribosome. *EMBO J* **17**, 7490-7497 (1998).

8 Rodnina, M. V., Gromadski, K. B., Kothe, U. & Wieden, H. J. Recognition and selection of tRNA in translation. *FEBS Lett* **579**, 938-942 (2005).

9 Johansson, M., Bouakaz, E., Lovmar, M. & Ehrenberg, M. The kinetics of ribosomal peptidyl transfer revisited. *Mol Cell* **30**, 589-598 (2008).

10 Pavlov, M. Y. *et al.* Slow peptide bond formation by proline and other N-alkylamino acids in translation. *Proc Natl Acad Sci U S A* **106**, 50-54 (2009).

11 Johansson, M. *et al.* pH-sensitivity of the ribosomal peptidyl transfer reaction dependent on the identity of the A-site aminoacyl-tRNA. *Proc Natl Acad Sci U S A* **108**, 79-84 (2011).

12 Johansson, M., Lovmar, M. & Ehrenberg, M. Rate and accuracy of bacterial protein synthesis revisited. *Curr Opin Microbiol* **11**, 141-147 (2008).

13 Strang, G. *Linear algebra and its applications.*, (1988).

14 Borg, A. & Ehrenberg, M. Determinants of the rate of mRNA translocation in bacterial protein synthesis. *J Mol Biol* **427**, 1835-1847 (2015).

15 Wang, J., Kwiatkowski, M. & Forster, A. C. Kinetics of Ribosome-Catalyzed Polymerization Using Artificial Aminoacyl-tRNA Substrates Clarifies Inefficiencies and Improvements. *ACS Chem Biol* **10**, 2187-2192, doi:10.1021/acschembio.5b00335 (2015).

16 Indrisiunaite, G., Pavlov, M. Y., Heurgue-Hamard, V. & Ehrenberg, M. On the pH dependence of class-1 RF-dependent termination of mRNA translation. *J Mol Biol* **427**, 1848-1860 (2015).
